# Insights on functionalized carbon nanotubes for cancer theranostics

**DOI:** 10.1186/s12951-021-01174-y

**Published:** 2021-12-16

**Authors:** Lu Tang, Qiaqia Xiao, Yijun Mei, Shun He, Ziyao Zhang, Ruotong Wang, Wei Wang

**Affiliations:** 1 State Key Laboratory of Natural Medicines, Department of Pharmaceutics, School of Pharmacy, China Pharmaceutical University, Nanjing, 210009 People’s Republic of China; 2 NMPA Key Laboratory for Research and Evaluation of Pharmaceutical Preparations and Excipients, China Pharmaceutical University, Nanjing, 210009 People’s Republic of China

**Keywords:** Carbon nanotubes, Intracellular targeting, Tumor microenvironment, Cancer theranostics, Targeted drug delivery

## Abstract

Despite the exciting breakthroughs in medical technology, cancer still accounts for one of the principle triggers of death and conventional therapeutic modalities often fail to attain an effective cure. Recently, nanobiotechnology has made huge advancement in cancer therapy with gigantic application potential because of their ability in achieving precise and controlled drug release, elevating drug solubility and reducing adverse effects. Carbon nanotubes (CNTs), one of the most promising carbon-related nanomaterials, have already achieved much success in biomedical field. Due to their excellent optical property, thermal and electronic conductivity, easy functionalization ability and high drug loading capacity, CNTs can be applied in a multifunctional way for cancer treatment and diagnosis. In this review, we will give an overview of the recent progress of CNT-based drug delivery systems in cancer theranostics, which emphasizes their targetability to intracellular components of tumor cells and extracellular elements in tumor microenvironment. Moreover, a detailed introduction on how CNTs penetrate inside the tumor cells to reach their sites of action and achieve the therapeutic effects, as well as their diagnostic applications will be highlighted.

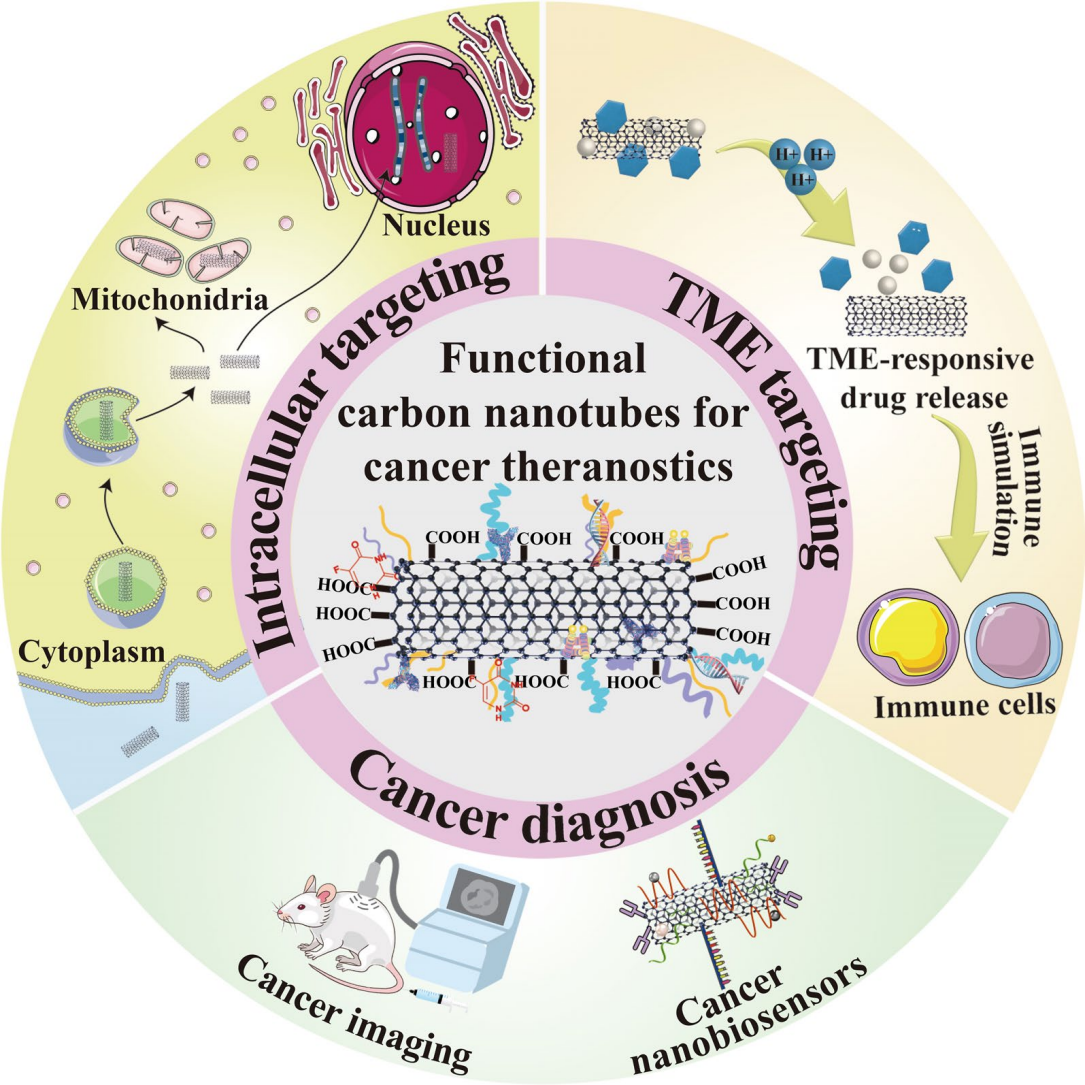

## Background

Cancer is one of the deadest diseases that causes an increasing mortality rates over the past decades [1]. This malignant illness is often characterized by an uncontrolled growth of cancer cells, which proliferates in an unlimited manner and invasively spreads to other organs and tissues throughout the body, disturbing the physiological function of normal cells and severely threating the people’s health and affecting their quality of life [2, 3]. Though the rapid development of treatment strategies like surgery, chemotherapy, radiotherapy, endocrine therapy, immunotherapy, phototherapy and gene therapy have greatly improved the current situation of cancer therapy and prolonged the patients’ survival time, these therapeutic modalities still face many challenges that hinder their broad application, especially for metastatic cancer treatment [4, 5]. For instance, surgery stalls cancer progression at the cost of significant pain and disfigurement of patients who have solid tumor [6]. Chemotherapy and radiotherapy are lack of specificity towards cancer cells, which can result in many unavoidable side effects and are able to promote drug resistance [7, 8]. Immunotherapy has achieved huge progress in improving clinical outcomes for cancer treatment with good selectivity, however, many patients suffer from systemic side effects like allergic reaction due to the dynamic and intricate interplay within immune system [9]. Other treatment strategies like phototherapy, gene therapy and endocrine therapy are highly specific and are only suitable to treat a part of cancer types because of their inherent characteristics [10]. Therefore, it is imminent to explore more safe and effective treatment strategies, or combine the current treatment methods in one regime to minimize their corresponding limitations.

Nanobiotechnology has emerged as a novel treatment opportunity against cancer in recent decades. The employment of nano-based drug delivery system (DDS) opened a new avenue to integrate multiple anticancer agents in one platform for more efficacious therapeutic outcome with reduced side effects and enhanced targeting ability [11–13]. Due to their unique potential for passive targeting through enhanced permeability and retention (EPR), high surface area for drug loading and modification, and prolonged plasma half-life, nanomedicine possesses numerous benefits over the conventional therapeutic modalities [14–16]. In this field, many nanocarriers like liposomes, micelles, reconstituted high-density lipoprotein (rHDL), metallic NPs, hybrid NPs, nanogel, nanoemulsion, black phosphorus, carbon-related nanomaterials, and other inorganic nanomaterials have been extensively explored in anticancer drug delivery both clinically and pre-clinically [17–19]. Among various nanocarriers, carbon nanotubes (CNTs) have arisen many researchers’ interest due to their multifunctional application possibilities in both cancer treatment and diagnosis. CNTs possess tiny tubular shapes comprising carbon atoms which are ordered to form a honeycomb nanostructure with exceptional physicochemical properties [20]. Generally, CNTs can be classified into either single-wall carbon nanotubes (SWNTs) or multi-wall carbon nanotubes (MWNTs) according to the sheet number of carbon atoms, both playing a critical role in cancer research with different properties [21].

**Fig. 1:**
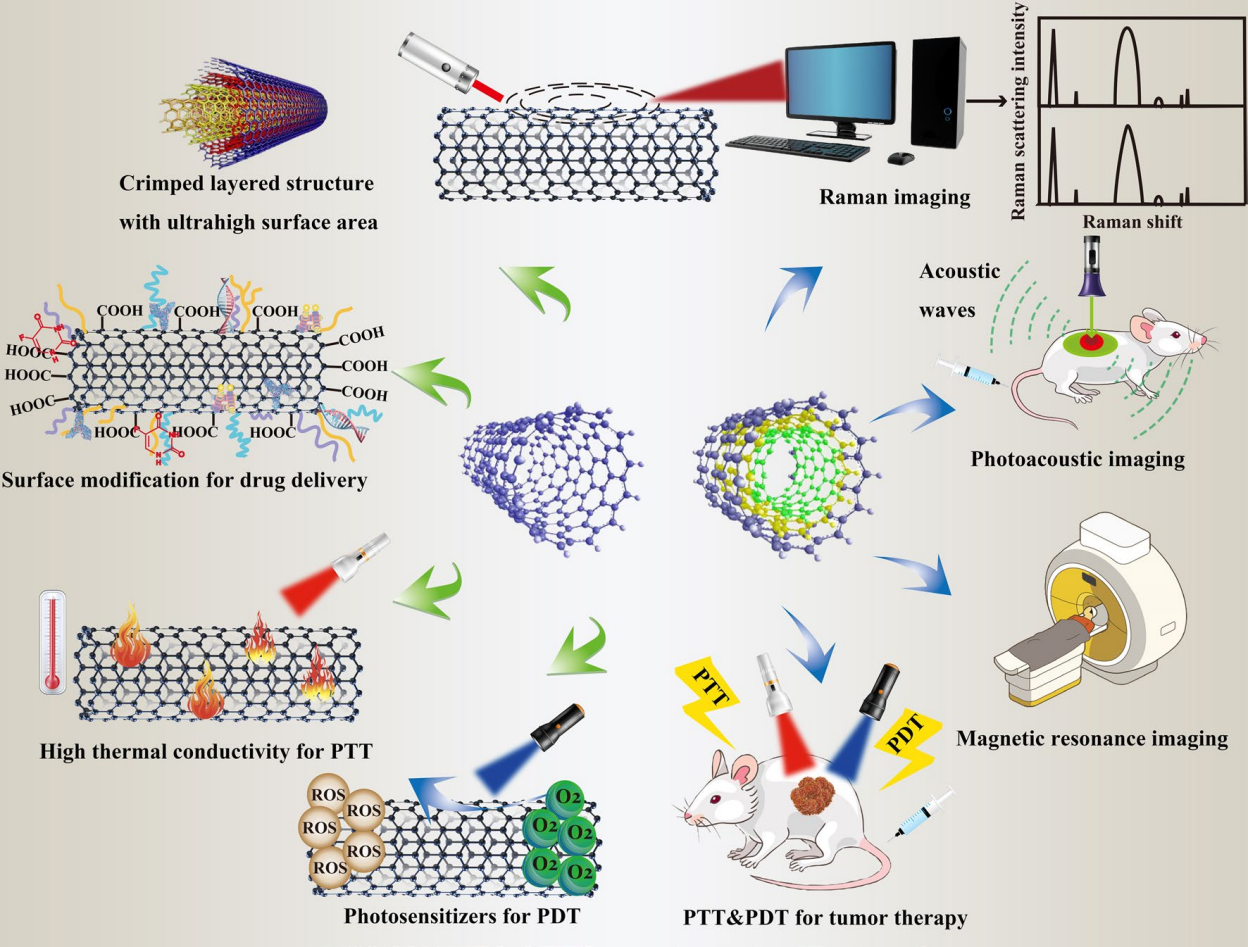
Schematic overview of the diverse applications of CNTs in cancer theranostics

Owing to their unique physiochemical features, CNTs are extensively studied in cancer theranostics with versatile application potentials [22]. For instance, the strong absorption of CNTs in near-infrared (NIR) regions enables their application in photothermal therapy (PTT). CNTs can transform the laser energy to acoustic signals and exhibit great resonant Raman scattering and photoluminescence in NIR region, which are all beneficial to their utilization in cancer imaging [23]. Besides, it has been reported that only a tiny amount of nanodrugs can be successfully delivered into solid tumors because of the biological obstacles and complicated tumor microenvironment (TME) that hinder their deep penetration [24]. Excitingly, numerous reports have demonstrated that CNTs can be taken up by various cell types due to their needle-like architecture, which promotes their tumor penetration as ideal vehicles for anticancer targeted drug delivery [25]. Moreover, CNTs can carry therapeutic agents to many intracellular targets like nucleus, mitochondria and cytoplasm, or target the TME components to disturb tumor cells’ living condition, both of which can result in an enhanced antitumor effect. Taken together, owing to their outstanding physicochemical characteristics, ultra-high surface for conjugation and drug loading, thermal conductivity, optical property, tumor penetration ability, CNTs are becoming rising stars in many biomedical fields including drug delivery, genetic engineering, imaging and bio-sensing to treat and diagnose a variety of cancers (Fig. 1) [26–28]. Of course, every coin has two sides, the distinctive structures of CNTs increase their hydrophobicity in water and leave them with inherent cytotoxicity [29]. Therefore, elevating hydrophilicity and reducing cytotoxicity through functionalizing CNTs with different chemical groups or biomolecules are of great significance to improve their safety and effectiveness for broad application in cancer therapy [30]. Thus, this review highlights the update progress of CNTs as promising drug delivery vehicles in cancer treatment. Different strategies used for their functionalization as well as their key roles in targeting various intracellular spots and TME constituents will be introduced. In addition, the recent advances of their application in cancer diagnosis will be outlined in detail. Therefore, compared to the previous reviews that summarized CNTs, the novelty of this paper is that we systemically introduce the theranostic applications of CNTs against many cancer types from the aspect of various therapeutic targets and emphasize the combination therapeutic modalities based on the physiochemical features of CNTs by using the fresh reported literatures [31–34].

## Functionalization of carbon nanotubes

**Fig. 2:**
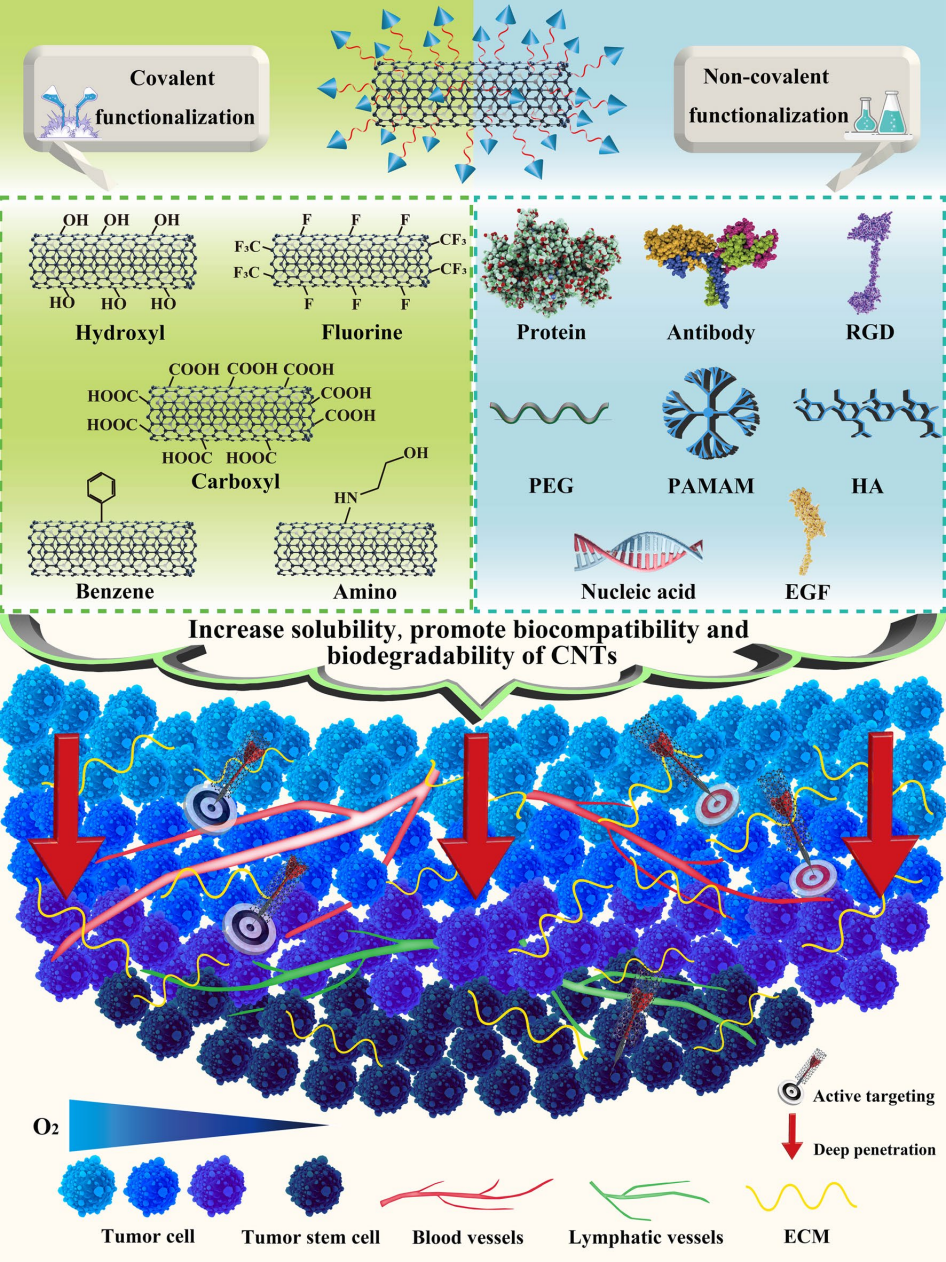
Schematic demonstration of different functionalization methods of CNTs. CNTs can be functionalized in covalent and non-covalent ways through various chemical groups, ligands and polymers to improve their solubility, biocompatibility and biodegradability for active drug targeting and deep tumor penetration

CNTs are initially insoluble and less dispersible substances, their inherent limitations such as poor solubility, agglomeration and toxicity severely hinder their wide applications. Hence, improving their natural properties through surface modification is of great necessity to acquire enhanced solubility, better biocompatibility, deeper penetration, and decreased cytotoxicity in biological systems [35]. Typically, there are two functionalization methods to modify CNTs, one is covalent, the other is non-covalent. Covalent modification of CNTs such as oxidation and carboxylation offers a stable platform for drug delivery, while non-covalent functionalization through Van der Waals interactions, π–π interactions, and hydrophobic interactions causes minimal damage to the surface of CNTs [36]. A lot of bioactive molecules such as antibodies and aptamers, various polymers and surfactants have been adopted to non-covalently functionalize CNTs to achieve increased biocompatibility and reduced toxicity (Fig. 2) [37]. Moreover, the biodegradability of raw CNTs are low because their hydrophobic interaction prevents enzymes from approaching it, however, their biodegradability can be improved through functionalization, which increases their solubility and produces defect sites to provide preferred binding sites for enzymes and promote enzymatic degradation [38]. For instances, defective sites on the carbon walls and ends of carboxylated- or nitrogen-doped MWNTs are more likely to bind to horseradish peroxidase (HRP), thus facilitating their enzymatic degradation [39]. Hence, the following sections mainly focus on the modifications of CNTs through various functionalized molecules. Different functionalization methods of CNTs are summarized in Table 1.

**Table 1:** Summary of the functionalization of CNTs through various molecules

Type of CNTs	Functionalized molecules	Effectiveness	Tumor model	Biocompatibility test	Refs.
SWNTs	PEG	Increase solubility, prevent particle aggregation, decrease side effect of the whole DDS	Gastric cancer	Show decreased toxicity towards normal tissue	[40]
Both SWNTs and MWNTs	PEI	Improve the solubility, homogeneity and dispersity, reduce the particle size, increase the positive charge to interact with siRNA	Cervical cancer	N/A	[41]
SWNTs	Hyaluronic acid	Enhance the serum stability and target CD44-overexpressing cancer cells, overcome the multidrug resistance	Ovarian cancer	Show no cytotoxicity towards normal tissue with no body weight drop in mice	[42]
MWNTs	Chitosan	Increase the water solubility and cell-penetrating ability, decrease the toxicity	Breast cancer	Show low cytotoxicity	[43]
MWNTs	PLGA	Improve the dispersity, reduce toxicity, provide attachment sites for drugs and tune the temporal release	Osteosarcoma	Show low cytotoxicity in normal cells	[44]
SWNTs	CD33 mAB	Recognize and specifically target the GNM-CD33^+^ cells	Glioblastoma	N/A	[45]
SWNTs	IGF1R mAB and HER2 mAB	Enable the multi-component to target IGF1R and HER2 surface receptors	Breast cancer	Exhibit negligible in vitro toxicity in normal cells	[46]
SWNTs	RGD peptide	Target α_v_β_3_-expressing cancer cells	Malignant melanoma	Induce little toxicity in vitro	[47]
SWNTs	EGF receptor	Achieve active targeting ability and enhance the uptake of drugs	Head and neck squamous carcinoma	Show negligible toxicity in vitro and in vivo	[48]

### Functionalization using polymer

Polyethylene glycol (PEG), a widely used polymer, was approved for pharmaceutical application by FDA, and has been utilized as an adjuvant material in drug delivery for many years due to its versatility and safety [49]. Therefore, PEG functionalization is a favorable choice to elevate the physiochemical characteristics of CNTs to achieve enhanced solubility and biocompatibility. To this aim, Taghavi et al*.* developed PEGylated SWNTs to carry DOX and Bcl-xL-specific short hairpin RNA (shRNA) for gastric cancer treatment, which exhibited an enhanced cancer cell death through the synergistic therapeutic effect of DOX and shRNA [40]. To achieve the active targeting purpose, the constructed PEGylated SWNTs were covalently linked to the nucleolin ligand AS1411 aptamer. PEG modification increased the solubility of SWNTs as well as prevented aggregation of particles and decreased the side effect of this DDS. According to the data of in vitro experiments, this combinational strategy through shRNA-based gene silence and chemotherapeutic agent DOX displayed excellent tumoricidal efficacy. In addition to PEG, polyethylenimine (PEI) is another widely used polymer that is suitable for gene delivery. As a polycation, PEI can incorporate with CNTs to reinforce the combining ability of CNTs with nucleic acid by π-stacking and electrical reaction [50]. Huang et al*.* modified both SWNTs and MWNTs with PEI by amination to obtain PEI-NH-SWNTs and PEI-NH-MWNTs [41]. As a result of PEI functionalization, both functionalized CNTs were capable of dispersing better in aqueous solution than SWNTs and MWNTs alone. Besides, CNTs modified with PEI could interact with siRNAs by electrical reaction due to CNTs’ positive charge on their surface, successfully delivering siRNAs into cells and resulting in higher transfection and gene silence efficiency. Hyaluronic acid (HA) is a linear macromolecular mucopolysaccharide with excellent biodegradability and biocompatibility and it is composed of alternatingly linked two saccharide units of N-acetylglucosamine and glucuronic acid [51]. CD44 receptor, the main binding receptor of HA, is usually overexpressed in many tumor types [52]. Therefore, HA is a promising material which can not only endow various nanoplatforms with active targeting ability to tumor tissue, but also enhance the biocompatibility of the whole DDS without additional toxicity during antitumor therapy. Bhirde et al. designed a CNT-based DDS by self-assemble method to actively deliver anticancer drug DOX to tumor site through the interaction between cholic acid-derivatized HA (CAHA) wrapped around semiconducting single-walled carbon nanotube (sSWNT) and CD44 receptors expressed on tumor cells [42]. According to the confocal video imaging results, DOX contained in this DDS could be effectively taken up by both drug-sensitive ovarian cancer cells OVCAR8 and resistant OVCAR8 (OVCAR8/ADR). In contrast, free DOX could only be taken up by drug-sensitive OVCAR8, but not by OVCAR8/ADR, demonstrating that CD44-targeted DDS was able to circumvent drug resistance during antitumor therapy and enhance the accumulation of drug in cancer cells. Other polymers like chitosan and polylactic-co-glycolic acid (PLGA) can also be adopted to modify CNTs [43, 44]. Chitosan is a bioactive polymer that derived from natural polysaccharides chitin, while PLGA is a synthetic polymer that has been approved by the FDA for medical application due to its great biodegradability and low toxicity [53]. Both modifications can greatly improve the inner properties of CNTs to achieve a better anticancer therapeutic efficacy.

### Functionalization using antibody

Monoclonal antibodies (mAbs) are artificial proteins with the similar functions like human antibodies and were firstly applied as antagonists of oncogenic receptor tyrosine kinases [54]. Nowadays, mAbs have been regarded as one of the promising substance for antitumor targeted therapy [55]. Several receptors, like cluster of differentiation-133 (CD133), human endothelial receptor 2 (HER2) and insulin-like growth factor 1 receptor (IGF1R), often show a overexpression on numerous types of cancer cells and cancer stem cells (CSCs) [56, 57]. Therefore, conjugating monoclonal antibody on CNTs opens a new era for antitumor treatment. CD133 is a common biomarker to detect CSCs and it serves as a prognostic indicator to guide tumor progression, remission and patient survival [58]. Thus, the application of CD133 monoclonal antibody (anti-CD133) can obviously enhance the targeting ability to CSC. Wang et al. combined SWNTs with anti-CD133-conjugated chitosan, forming anti-CD133-CS/SWNTs for glioblastoma (GBM) treatment [45]. In vitro experiment showed that this nanosystem exhibited excellent targeting ability to GBM-CD133^+^ cells, and could effectively eliminate the cancer cells upon NIR irradiation without damaging surrounding tissues. Besides, the combination of monoclonal antibody with PTT displayed good inhibitory effects on tumor invasion through preventing the self-regrowth of GBM-CD133^+^ cells in vitro. Integrated molecular targeting is another approach that can be combined with CNTs during antitumor therapy. IGF1R and HER2 are two surface markers that both overexpress in various cancer types, exhibiting great research values [59]. Shao et al. designed a nanosystem consisted of SWNTs, IGF1R monoclonal antibody (anti-IGF1R) and HER2 monoclonal antibody (anti-HER2), which could target IGF1R and HER2 in tumor cells [46]. In vitro experiments based on breast cancer cell lines showed that antibody-modified SWNTs could internalize into cancer cells more effectively through the interaction between specific antibodies and their corresponding antigens. In addition, the integration of PTT with the aforementioned mAB-therapy could significantly destroy the breast cancer cells in vitro, indicating that antibody-functionalized SWNTs combined with their intrinsic optical properties could provide an effective way for anticancer treatment.

### Functionalization using RGD peptide

Arginylglycylaspartic acid (RGD) is a small peptide that serves as an ideal ligand for most integrin types expressed on various cancer cells [60]. Thus, combining RGD peptide onto the surface of CNTs can improve the active targeting ability of the DDS as well. Integrin α_ν_β_3_, a common form of integrin, is overexpressed on many cancer cells and contributes a lot to tumor progression [15]. Koh et al. constructed a RGD peptide modified, carboxylic acid functionalized SWNTs with the encapsulation of the topoisomerase I inhibitor camptothecin (CPT) (CPT@*f-*CNT-RGD) to treat malignant melanoma [47]. According to the results of cell viability assay, 3D-cultured cells model, and western blot assay, the human malignant melanoma cell A375 incubated with CPT@*f*-CNT-RGD exhibited the lowest cell viability and expressed the highest level of apoptosis-related proteins such as caspase-3 and Bax. In addition, the in vivo distribution studies showed that CPT@*f-*CNT-RGD could localize in tumor sites to a great extent, confirming the excellent targeting effect of this nano DDS.

### Functionalization using epidermal growth factor

Epidermal growth factor (EGF) is the ligand of EGF receptor (EGFR) overexpressed on the surface of various tumor cells [61]. Therefore, combining CNTs with EGF endows CNTs with tumor-targeting ability and can be regarded as another method to improve therapeutic efficacy of anticancer treatment. Cisplatin is a classic anticancer drug with broad spectrum to treat many cancer types [62]. Bhirde et al. conjugated both cisplatin and EGF to the surface of oxidized SWNTs, which endowed the nanosystem with active targeting ability through the interaction between EGF and EGFR overexpressed on tumor cells for enhanced antitumor efficacy of cisplatin [48]. In vitro and in vivo studies based on head and neck squamous carcinoma model showed that EGF/cisplatin/CNTs exhibited the best active targeting ability and the strongest cytotoxicity to tumor cells, which could inhibit the tumor growth to the greatest extent compared to other groups without EGF modification, further demonstrating that the active targeting ability of the whole DDS could be enormously improved with the existence of EGF, while the potential adverse effects due to the lack of specificity could be accordingly avoided.

## Carbon nanotubes in cancer treatment

CNTs have been widely investigated in targeted drug delivery for cancer therapy due to their distinguished properties. CNTs can not only serve as drug carriers to deliver various anticancer cancer agents, but also function as excellent mediators in phototherapy because of their inherent optical features. The multifunctionality of CNTs enable their multiple applications in treating various cancers. Currently, many anticancer therapeutic strategies are aiming at targeting tumor cells and the environment where they survive. Targeting tumor cells can effectively destroy their parenchyma in a direct way, while targeting TME can inhibit their growth and metastasis through disturbing their living conditions, which also kills tumor cells indirectly. Therefore, the following sections will give a detailed introduction on how CNT-based nano drug delivery systems target various therapeutic spots including intracellular targeting spots of tumor cells and the extracellular components in TME for cancer therapy (Table 2).

**Table 2:** Application of CNT-based DDS in targeting various intracellular therapeutic spots and TME components

Targeting spot	Drug delivery system	Therapeutic modality	Tumor model	Effectiveness	Biocompatibility test	Refs.
Nucleus	PEG-SWNTs-DOX	Photothermal therapy + Chemotherapy	Breast cancer	Increase delivery efficiency, promote the accumulation and localization of DOX inside the nucleus, cause effective cancer cell death	N/A	[63]
Nucleus	SWNTs-carrier	Chemotherapy	Colorectal cancer	Achieve targeted therapy and controlled drug release	Show good biocompatibility	[64]
Nucleus	*f*-SWNTs-p53 plasmid complexes	Gene therapy	Breast cancer	Transport the target gene into the nucleus effectively, induce apoptosis strongly	N/A	[65]
Cytoplasm	Chim/PEI/5-FU/CNT nanoparticles	Gene therapy + Chemotherapy	Gastric cancer	Achieve targeted delivery and silence the drug-resistant gene, promote the apoptosis of drug-resistant cancer cells	Show negligible in vivo toxicity, and none of the mice dead after treatment with no statistically significant difference in body weight between the groups	[66]
Cytoplasm	MWNTs/Sor/siRNA	Gene therapy + Chemotherapy	Liver cancer	Enhance the release of sorafenib and improve siRNA stability, display significant antitumor effect	N/A	[67]
Cytoplasm	SWNT-HIF-1α siRNA complexes	Gene therapy	Pancreas cancer	Transfect and induce the RNAi response, effectively suppress tumor growth	Negligible toxic effect in vitro and in vivo	[68]
Cytoplasm	oxDWNT-siRNA	Gene therapy	Prostate cancer	Release siRNA into the cytoplasm to suppress survivin protein synthesis, directly cause cancer cell apoptosis	Show good biocompatibility	[69]
Mitochondria	MWNT-Rho (PtBzt + BP)	Chemotherapy	Ovarian cancer	Increase the selectivity of platinum-based chemotherapy, minimize the side effects	N/A	[70]
Mitochondria	PEG-CNTs-ABT737	Chemotherapy	Lung cancer	Improve the mitochondrial targeting of lung cancer cells, cause cancer cell apoptosis	Show lower cytotoxicity in NHFB normal cells than A549 lung cancer cells	[71]
Mitochondria	P-D-CS-CNTs	Photothermal therapy	Bladder cancer	Enhance mitochondrial targeting, induce the ROS burst, result in mitochondrial damage and cell death	N/A	[72]
Mitochondria	PL-PEG-SWNT	Photoacoustic therapy	Breast cancer	Transform pulse laser energy into sound energy, bomb the mitochondria into dysfunction and trigger mitochondrial outer membrane permeabilization	Show low toxicity without epidermis injury	[73]
Extracellular matrix	MWNTs	Photothermal therapy	Epidermoid carcinoma	Significant soften tumors together with volume reduction, induce the destruction of collagen and cell damage	N/A	[74]
Cancer stem cells	SWNT-Raw and SWNT-COOH	Chemotherapy	Osteosarcoma	Specifically bind to TGFβ1-induced activation of TGFβR1 and suppress its downstream signaling, decrease the OSCs population	Exhibit no obvious toxicity to normal cells	[75]
Tumor vasculature	DOX/CD-CNT and CUR/CD-CNT	Photothermal therapy + Chemotherapy	Hepatocellular carcinoma	Enhance drug entrapment efficiency and achieve sustained release of both drugs	Cause minimal damage to normal cells	[76]
Tumor vasculature	iRGD-PEI-MWNT-SS-CD/pAT2	Chemotherapy	Lung cancer	Promote the cellular uptake and transfection efficiency, inhibit angiogenesis, suppress tumor growth significantly	Not induce obvious tissue damage or inflammatory cell infiltration, not affect blood, hepatic or kidney function in mice	[77]
PD-1/PD-L1	Rg3-CNT	Immunotherapy	Triple-negative breast cancer treatment	Inhibit PD-1/PD-L1 axis and the TNBC cell growth	N/A	[78]
Immune cells	MWNTs-DOX and MWNTs-CpG	Immunotherapy + Chemotherapy + Phototherapy	Melanoma	Inhibit tumor growth, enhance the number of CD4^+^ and CD8^+^ T cells, promote TAM shifting, reduce the number of Tregs in TME	Show non-toxicity to the organs (liver, spleen, kidneys, heart and lungs) in mice	[79]

### Carbon nanotubes for intracellular targeting

**Fig. 3:**
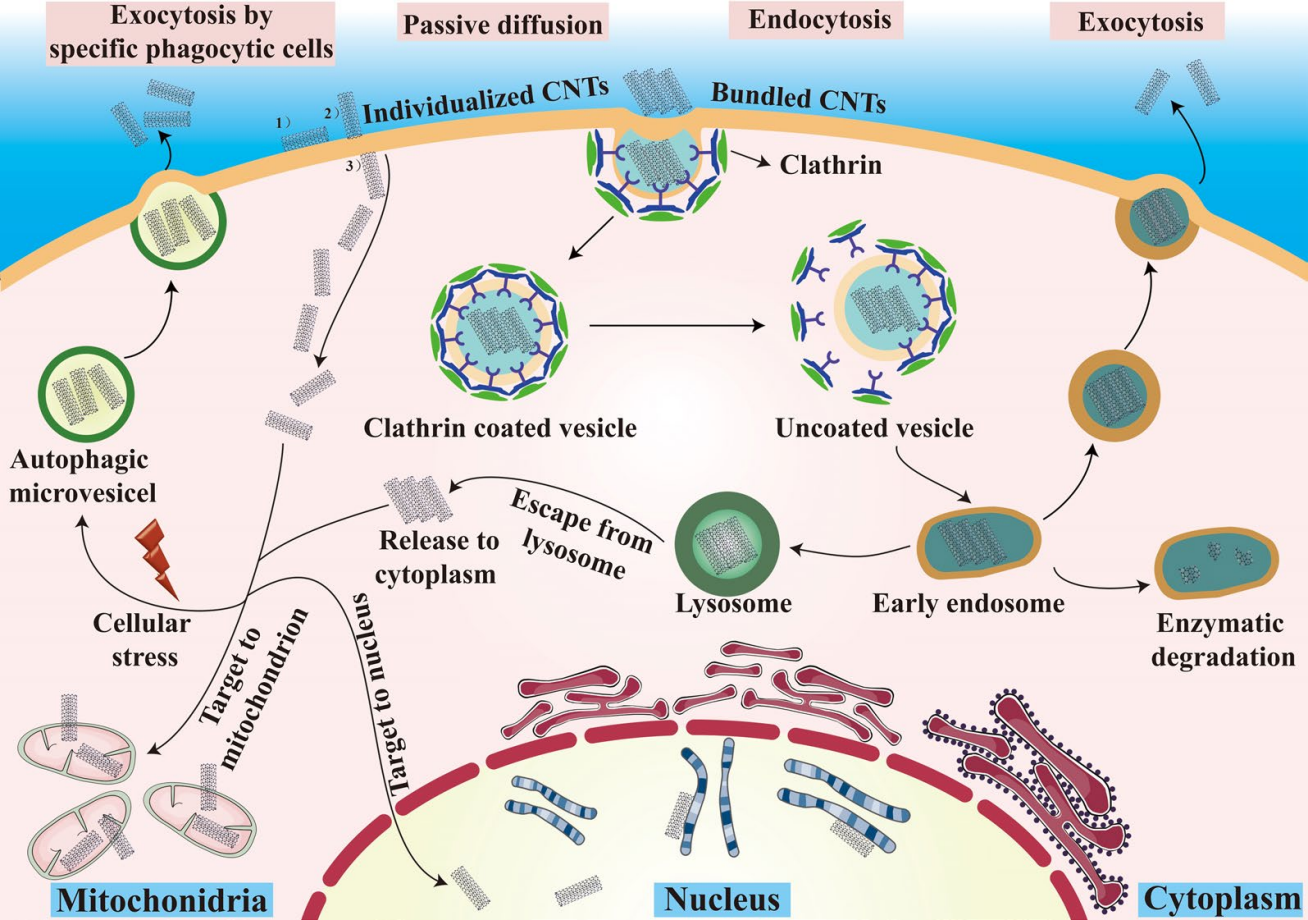
Schematic demonstration of intracellular targeting spots, uptake and cellular fate of CNT-based DDS

Intracellular drug delivery has gained a considerable attention in cancer therapy. By this delivery strategy, anticancer agents can be delivered to particular intracellular targeting spots, which not only maximizes their therapeutic efficacy, but also reduces their corresponding toxicity [80]. The most popular intracellular targets include nucleus, cytoplasm, and mitochondria. Nucleus acts as a regulatory center for cellular genetics and plays a key role in cell metabolism, growth, and differentiation [81]. Cytoplasm is composed of cytoplasmic matrix, endomembrane system, cytoskeleton and inclusions, serving as the main site of cellular activity [82]. Mitochondria are vital intracellular organelles that carry out a variety of functions and are involved in many metabolic pathways. In addition, mitochondria are responsible for the synthesis of the energy-rich molecule adenosine triphosphate (ATP), which are required for many cellular processes [83]. Therefore, these three intracellular elements can be used as potential therapeutic targets for precise cancer treatment. Nanocarriers offer a number of advantages for intracellular drug delivery, by which therapeutic agents can be specifically transported to their sites of action with much lower drug concentration to elicit a better effect than drugs administered in the free form. CNTs are attractive carriers to deliver both biomolecule and small molecule anticancer drugs. Through appropriate functionalization, the biological performance of CNTs can be intensively elevated with reduced toxicity, which benefits their application in anticancer drug delivery. Thus, the following sections give an introduction of the recent examples in CNT-mediated anticancer intracellular targeting via various delivery strategies (Fig. 3).

#### Nucleus targeting

The nucleus of cells serves as the regulation center to control the proliferation and metabolism of cells, manage cell cycle and active certain genes, therefore, nucleus is considered as a desired therapeutic target for malignant tumor treatment. Nucleus targeting or nucleus membrane-penetrating nanoparticles are expected to provide a more effective approach in cancer therapy. Oh et al*.* constructed a DDS that carried DOX with PEGylated SWNTs for combined NIR-irradiated PTT and chemotherapy [63]. DOX is a widely adopted topoisomerase inhibitor in chemotherapy, however, the toxic side effects generated by DOX due to its non-specific distribution and high administration dosage significantly limit its further application. Therefore, SWNTs with high surface area provide an approach for the efficient drug delivery of DOX. In this study, the synthesized PEG-SWNTs-DOX could lead to effective breast cancer cell death in comparison to the single treatment with DOX or PTT alone. Besides, the released DOX from carrier could accumulate inside the cancer cells at high concentration to effectively localize into their nucleus, demonstrating the excellent potential of PEG-SWNTs-DOX for breast cancer treatment. Lee et al. designed a nanotube system that used SWNTs to conjugate with an EGFR inhibitor cetuximab and topoisomerase I inhibitor SN38 to realize the precise treatment of EGFR over-expressing colorectal cancer [64]. SN38 was covalently linked to SWNTs via carbamate bond that could be gradually broken up by carboxylesterase enzyme (hCE) in lysosomes, therefore, SN38 was controllably released inside the cells. In vitro results obtained from different colon cancer cell lines showed that SN38 was able to entry the nucleus after dissociation from the SWNT-carrier, while leaving the carrier in the cytoplasm, which confirmed that SWNTs were excellent vehicles for the targeted transportation of SN38 to nucleus.

**Fig. 4:**
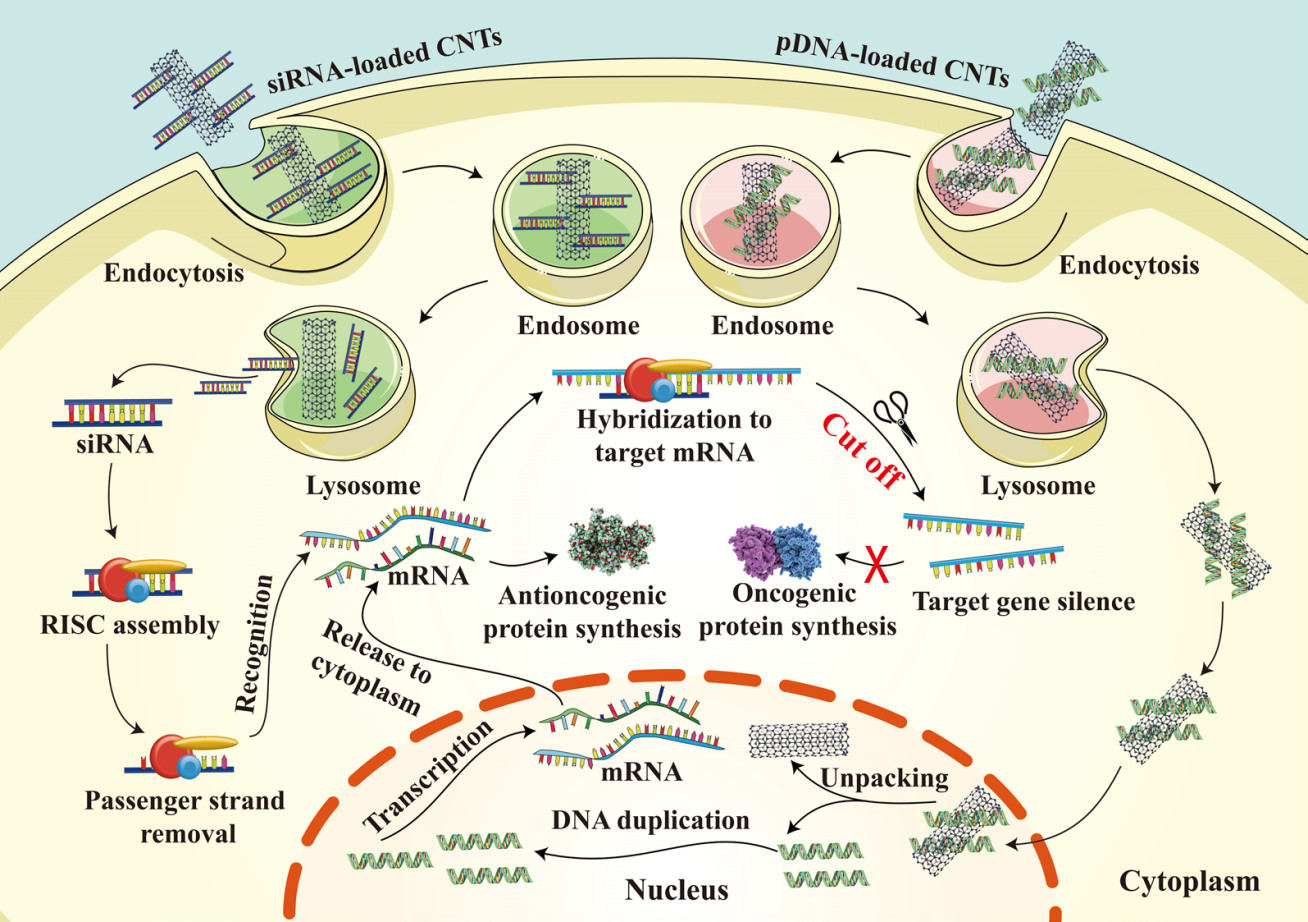
Gene therapy mechanisms of siRNA-loaded CNTs and pDNA-loaded CNTs in cancer treatment

Gene therapy is an emerging area with the goal to rectify genetic disorders at the molecular level. Gene therapy mainly includes the transport of genes with plasmid DNA (pDNA) to elevate downregulated genes and change mutated genes, or through the application of small interfering RNA (siRNA), micro RNA (miRNA) and shRNA to decrease the protein expression by interference in RNA level (Fig. 4) [84].

A satisfactory gene therapy effect is dependent on the vector that protects exogenous DNA from degradation and inactivation, or avoids lysosomal degradation within the cells. Therefore, an effective delivery vehicle plays a key role in achieving successful gene therapy outcomes. CNTs, a class of nonviral gene vector, have attracted increasing attention in gene delivery. CNTs are reported to have an interaction with many biological constituents and are able to be effectively internalized in various cell types via distinct mechanisms [85]. In addition, unlike viral vectors, nonviral vectors tends to display a better immunogenic performance, which will not cause severe immunogenic side effects to the body. Therefore, Karmakar et al. used the oncogene suppressor protein 53 (p53) gene as the mode gene and functionalized SWNTs as the vector for breast cancer treatment [65]. p53 is an important anti-oncogenic gene which involves in the DNA repair procedure or trigger cell suicide when DNA damage is irreparable [86]. In vitro experiments verified the feasibility of transporting *f*-SWNTs-DNA complex with p53 plasmid into MCF-7 breast cancer cells, which demonstrated that *f*-SWNTs-p53 plasmid complex could enter into the cytoplasm of cancer cells via endocytosis and then arrive at their nucleus, where the p53 plasmid could be separated from the *f*-SWNTs vector due to the pH change between nucleus and cytoplasm. Finally, the p53 gene got transcription and translation to activate the apoptosis pathways in the cytoplasm. Taken together, all these aforementioned studies evidenced that *f*-SWNTs are promising gene vectors that could transport the target gene into the nucleus with good effectiveness.

#### Cytoplasm targeting

Cytoplasm is a gel-like scaffold composed of various macromolecules, organelles, cytoskeletal networks, and cytosol with a highly organized and heterogeneous architecture due to the variety of its constituents [87]. Cytoplasm participates in a number of important cellular activities such as cell division and polarization, and serves as the main site for cellular activity [88]. As is mentioned above, CNTs can be applied as effective vectors for gene therapies due to their capability to efficiently enter cells. Apart from gene insertion into the host genome via pDNA, silencing the pathogenic gene expression through siRNA can also achieve great gene therapy efficacy [89]. Moreover, there is no need for RNA to integrate into the host genome, therefore, RNA-based gene therapy exhibits a better safety profile than pDNA-based gene therapy. In addition, siRNA achieves its sites of action in the cytoplasm, therefore, cytoplasm is an essential place for RNA interference (RNAi) (Fig. 4).

An example carried out by Chen et al*.* constructed a nano DDS based on CNTs to effectively deliver chemotherapeutic agent 5-FU and for the treatment of drug-resistant gastric cancer with peritoneal dissemination [66]. Aptamer-siRNA chimera could specifically bind to the cancer cells to enable the whole DDS with active targeting ability for precise transportation of 5-FU. In vitro experiments obtained from MKN45 gastric cancer cell line revealed that this constructed DDS was able to facilitate 5-FU-resistant gastric cancer cell apoptosis as well as inhibit their invasion and proliferation, which in turn enhanced the effect of chemotherapy. In addition, the drug-resistant gene was effectively silenced through therapeutic siRNA, which all evidenced that this CNT-based nanocarrier could function as a great vehicle to deliver siRNA and chemotherapeutics. Similarly, Wen et al*.* used MWNTs to co-deliver the multi-target kinase inhibitor sorafenib (Sor) and EGFR siRNA for liver cancer treatment [67]. Sor is a widely applied anticancer drug in clinic because it can suppress cancer-associated pathways. EGFR is overexpressed in many cancer types, therefore, specific inhibition of EGFR expression can induce cell apoptosis through reducing the activity of EGF [90, 91]. As a result, combinational treatment strategy of Sor and EGFR targeting through CNT-based DDS is an ideal therapeutic approach for liver cancer treatment. The obtained nanocomposite could increase the release of Sor and enhance siRNA stability by protecting it from degradation. In vitro results demonstrated that this nanocomposite could significantly inhibit the replication of liver cancer cells and induce their apoptosis compared to the control group. In vivo experimental data further evidenced that treatment with this nanocomposite could obviously decrease the tumor volume and weight in tumor-bearing mice, indicating its superior effectiveness for liver cancer treatment. Apart from the examples mentioned above, Bartholomeusz et al*.* used SWNTs to deliver hypoxia-inducible factor 1 alpha (HIF-1α) siRNA into pancreas cancer cell to inhibit the expression of HIF-1α [68]. As hypoxia is one of the most characteristic features of rapidly proliferating solid tumor, which makes them become aggressive and difficult to treat, therefore, tumor hypoxia is regarded as a potential therapeutic target and a lot of researches have already reported that HIF-1α inhibition can suppress tumor growth by reducing its activity [92]. This nanocomplex could efficiently achieve the goal of RNAi and effectively suppress tumor growth, further demonstrating the key role of CNTs as a good siRNA delivery platform. In addition to SWNTs and MWNTs, double-walled carbon nanotubes (DWNTs), which bridge the gap between SWNTs and MWNTs could also be applied to delivery siRNA. Neves et al. loaded survivn siRNA by polypeptide (Poly(Lys: Phe)) coated DWNTs for prostate cancer treatment [69]. Due to the nano-needle structure, DWNTs could efficiently enter mammalian cells and was able to escape from lysosomes and release siRNA into the cytoplasm to suppress survivin protein synthesis, directly causing cancer cell apoptosis. To summarize, CNT-based nano DDS is a promising tool to carry siRNA into cytoplasm for efficient gene therapy.

#### Mitochondria Targeting

Mitochondria, one of the most important organelles within the cells, are around 1 µm in diameter with variable length. Mitochondria are well-known to participate in energy metabolism such ATP synthesis [93]. Besides, they are pivotal cellular organelles involved in the generation of ROS, and regulation of apoptosis, autophagy and necroptosis [94]. Accumulating evidence have already demonstrated that mitochondrial bioenergetics, biosynthesis and signaling play a fundamental role in tumorigenesis, and mitochondria are indispensible energy supporter for highly proliferating cancer cells, thus, mitochondria are regarded as important therapeutic targets in cancer treatment [95]. However, the lack of efficient delivery vehicle that can load therapeutic molecules with specific targeting ability and internalization capability limits the therapeutic strategy based on mitochondrial targeting. Fortunately, CNTs are capable of being internalized by various cancer cells, and can target mitochondria by mitochondrial targeting ligands such as mitochondriotropics [96]. In addition, CNTs possess excellent optical properties and can be applied in PTT, photoacoustic therapy (PAT) and thermoacoustic therapy (TAT) to directly destroy the mitochondria of cancer cells, thus causing obvious killing effect on cancer cells.

**Fig. 5:**
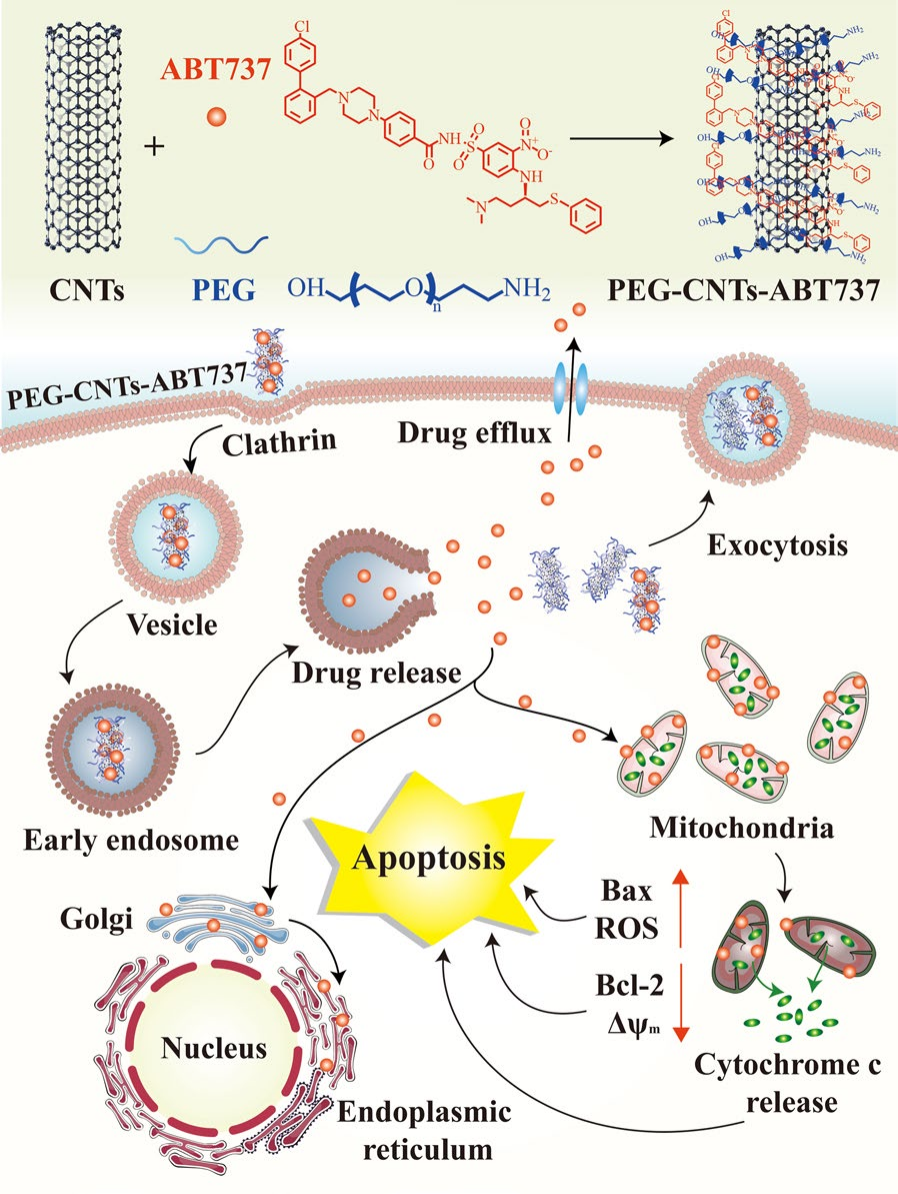
Schematic demonstration of the construction and working mechanism of PEG-CNTs-ABT737 nanodrug for anticancer therapy

Chemotherapy remains to be the most widely adopted strategy to treat various cancers, however, due to their non-specificity, indiscriminate destruction of normal cells, and the development of multidrug resistance, chemotherapy often causes undesirable side effects and the application of chemotherapy is still limited [97]. Benefiting from their attractive properties, CNT-based DDS have been explored and designed for the precise delivery of chemotherapy drugs to mitochondria. Yoong et al*.* functionalized MWNTs with fluorescent rhodamine-110 with mitochondrial targeting ability (MWNTs-Rho) to co-encapsulate a chemo-potentiator 3-bromopyruvate (BP) and platinum (IV) prodrug (PtBz), constructing a DDS called MWNT-Rho (PtBzt + BP) for cancer treatment [70]. PtBz, which were entrapped within the core of hydrophobic MWNTs, could be transformed into platinum (II) by glutathione, which turned to be hydrophilic species and could be released from MWNTs [98]. After reaching mitochondria of the cancer cells via active targeting effect of Rho-110, platinum (II) could covalently crosslink and inhibit the transcription and replication of mitochondrial DNA (mtDNA) that encodes 13 polypeptides essential for mitochondrial function, eventually causing cell apoptosis [99]. In vitro experiments showed that co-incubation of PtBz with BP exhibited significant cytotoxicity to human ovarian cancer cells. In addition, owing to the unique shape of CNTs and the lipophilic cationic nature of Rho-based dyes, MWNTs-Rho (PtBz + BP) could be effectively internalized into cells and target mitochondria, which mitigated the poor selectivity of platinum-based chemotherapy and minimized the side effect. Although targeting mitochondria is an effective method to induce cancer cell apoptosis, the limited targetability towards mitochondria impedes its clinical effectiveness for cancer treatment. To address this issue, Kim et al*.* designed a ABT737-loaded PEGylated CNT-based nano DDS (PEG-CNTs-ABT737) with enhanced mitochondrial targetability towards lung cancer cells [71]. ABT737 is a small molecule inhibitory compounds that can suppress B-cell lymphoma-2 (Bcl-2) proteins which is an anti-apoptotic protein for efficacious cancer treatment [100]. As is illustrated in Fig. 5, PEG-CNTs-ABT737 nanodrug was able to escape from endosome and could deliver ABT737 into the mitochondria of A549 lung cancer cells, which resulted in the mitochondrial accumulation of nanodrug to cause cancer cell apoptosis [101] (Fig. 5).

Apart from the application as drug carriers, CNTs can be utilized in PTT due to their superior NIR absorption efficiency [102]. Mitochondria was reported to be sensitive to hyperthermia and can be considered as targets for PTT [103]. Wang et al*.* fabricated a nanosystem based on SWNTs for the PTT of bladder cancer [72]. In vitro experiments showed that this nanosystem could destroy the mitochondria of MB49 murine bladder cancer cells after mild hyperthermia produced by SWNTs-mediated PTT, which led to the burst of ROS from damaged mitochondria to induce cell death. Although PTT is one of the emerging therapeutic modalities with non-invasive or slightly invasive properties for cancer treatment, it can also inevitably induce partial skin damage. Therefore, photoacoustic therapy (PAT), another innovative phototherapy strategy, is adopted to selectively eliminate tumor cells without causing epidermis injury [104]. The dose of laser and treatment model are two main differences between PTT and PAT, PTT usually irradiates the local tissue continuously with a higher laser dose, while PAT can be generated through a lower laser dose by transducing light energy into acoustic pressure [105]. For example, Zhou et al. applied PAT for mitochondria-targeting destruction by using the photoacoustic transducer property of SWNTs with the selective absorption of 1064 nm NIR pulse laser [73]. SWNTs were able to transform pulse laser energy into sound energy that could bomb the mitochondria into dysfunction and trigger mitochondrial outer membrane permeabilization (MOMP) to cause intrinsic apoptosis [106]. In vitro experiments showed SWNTs-based treatment accompanied with laser irradiation could induce obvious cell death in EMT6 mouse mammary tumor cells compared to the control group, confirming that the sound energy stimulated by SWNTs was able to trigger a much higher level of apoptotic cell death due to the initiation of cancer cell apoptosis through mitochondrial pathway.

### Carbon nanotubes for tumor microenvironment targeting

**Fig. 6:**
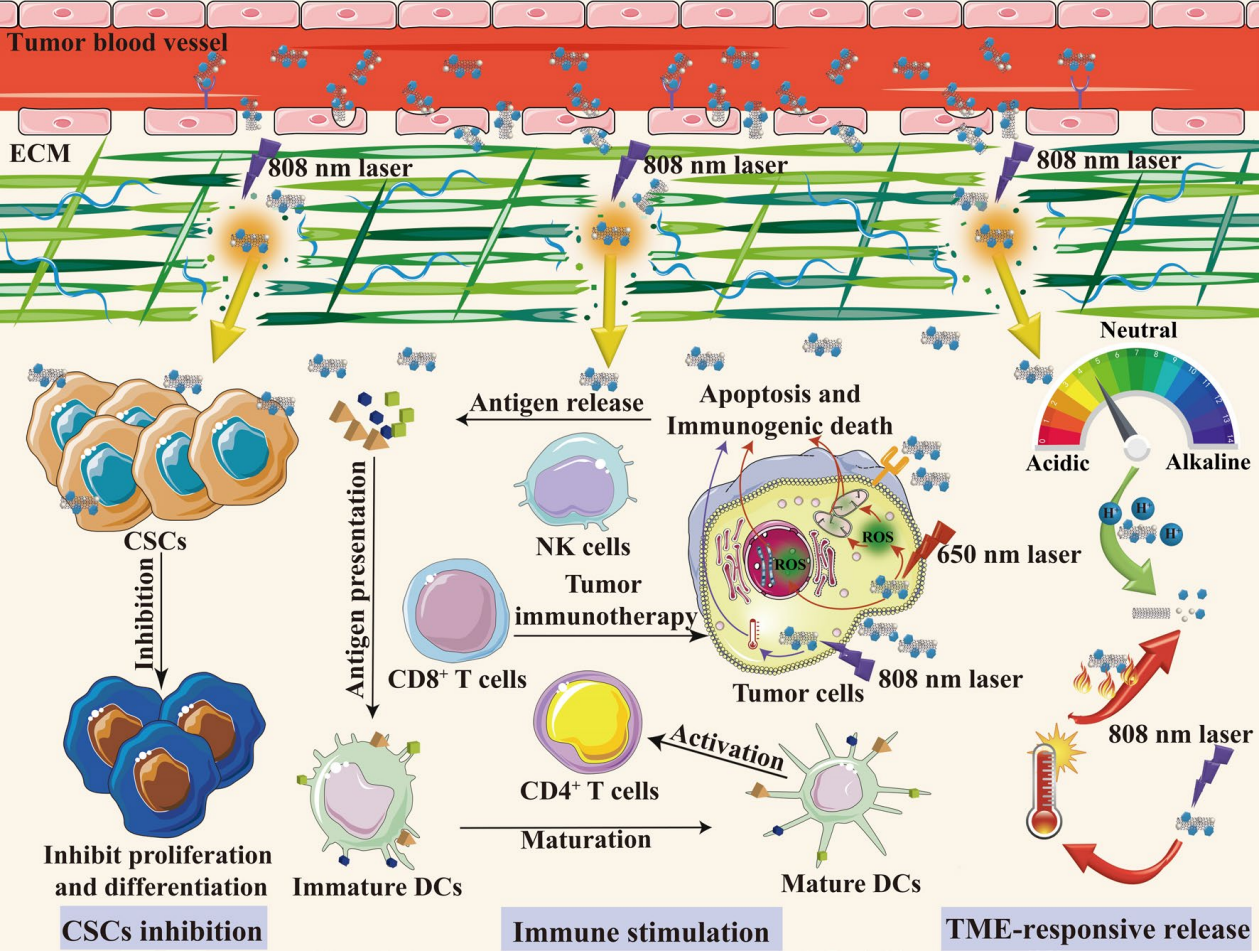
Schematic graph of CNT-based DDS in TME targeting

A number of researches have clearly revealed the critical role of TME in tumorigenesis. TME is composed of a complicated system involving various cell types including endothelial cells, pericytes, immune cells, CSCs, cancer-associated fibroblasts (CAFs) and the dense extracellular matrix (ECM) [107, 108]. The TME-constituting cells have a dynamic interplay with the cancer cells, serving as the soil for tumor initiation, development, invasion, migration, and therapy response [109]. Therefore, TME exhibits great research and clinical importance in cancer therapy. Recent decades have witnessed the huge progress of employment of nanotechnology in cancer therapy. Except for the tumor cells, nano-based DDSs can be designed as demand to accurately target TME elements and remodel the TME for tumor regression [11]. Owing to their unique properties, multifunctional CNT-based nano DDSs have shown outstanding ability in targeting the components of TME [110]. Thus, this section highlights the recent applications of CNTs in TME-based cancer therapy, various strategies including ECM remodeling, tumor vasculature targeting, immune stimulation, CSCs inhibitory and TME-responsive drug release will be outlined (**Fig. ****6**).

#### Extracellular matrix remodeling

ECM is commonly defined as the non-cellular constituents of three-dimensional macromolecular scaffold that supplies biochemical and structural support for its cellular constituents [111]. Fundamentally, ECM is a dense environment mainly composed of collagen, proteoglycans, laminin, and fibronectin. This diverse composition of ECM endows it with distinct bio-physicochemical properties to regulate various cellular processes within the TME [112]. Besides, several studies have demonstrated that ECM is the first barrier before tumor migration, metastasis and proliferation, and its dysregulation can accelerate tumor progression [113]. Thus, understanding the interplay between tumor cells and ECM as well as investigating potential therapeutic strategies through targeting ECM components will open a new era for anticancer therapy.

Collagen is considered as the basis of ECM structure and is the most abundant protein discovered in human tissue [114]. As tumor cells are usually embedded in a dense ECM comprising cross-linked collagen, hyaluronic acid, and different proteins, the rich matrix composition of these substances impedes the deep penetration of therapeutic agents to tumor cells, thus greatly limiting their therapeutic efficacy [115]. Therefore, reduction of collagen density and tumor stiffening result from the abnormal organization of ECM are possible approaches to enhance the penetration and accumulation of drug at tumor sites. Marangon et al*.* studied the effects of local hyperthermia produced by MWNTs on epidermoid carcinoma microenvironment to explore the relationship between tumor stiffness evolution and tumor progression [74]. Physical strategies using hyperthermia or photothermia represent as an alternative strategy to ablate the components of ECM. In their study, two conditions of heating including mild hyperthermia (43 °C for 15 min) and thermal ablation (52 °C for 3 min) were generated by light-exposed MWNTs through tuning NIR irradiation power to explore the effects of nanomaterial-mediated heating on ECM remodeling. According to their observation, the irradiated tumors showed an obvious softening accompanied with volume decrease over the days after PTT compared to the non-irradiated group. In addition, with the imaging of second-harmonic generation (SHG) microscopy and two-photon excitation fluorescence (TPEF) microscopy, MWNTs-mediated PTT was shown to induce the destruction of collagen and cell damage, indicating that nanohyperthermia was an alternative approach to decrease tumor stiffening and remodel the dense ECM for better drug penetration.

#### Cancer stem cell inhibition

CSCs are a subpopulation of cancer cells which are characterized by highly tumorigenic and intrinsically drug-resistant. They are able to self-renew and differentiate into diverse tumor cell types [116]. Accumulating researches have evidenced the key role of CSCs in modulating TME, which involves in different stages of tumorigenesis including tumor growth, metastasis and recurrence [117, 118]. In addition, growing evidence demonstrated that CSCs are capable of withstanding classical therapeutic modalities like chemotherapy and radiotherapy through various mechanisms such as elevated expression of drug transporters, enhanced DNA repair ability and maintenance of a slow dividing state, which result in the development of drug resistance [119]. Besides, owing to their good tumorigenic and survival abilities, a group of CSCs can probably survive under conventional cancer treatment strategies and are able to seed new tumors in second generation, leading to the quick relapse of cancer [120]. Therefore, CSCs are regarded as potential target spots within the TME for cancer therapy. As CNTs are able to integrate molecular targeting and PTT in one platform, thus, CNTs-based therapeutic strategies are promising methods to destroy CSCs as well as induce their apoptosis through hyperthermia.

**Fig. 7:**
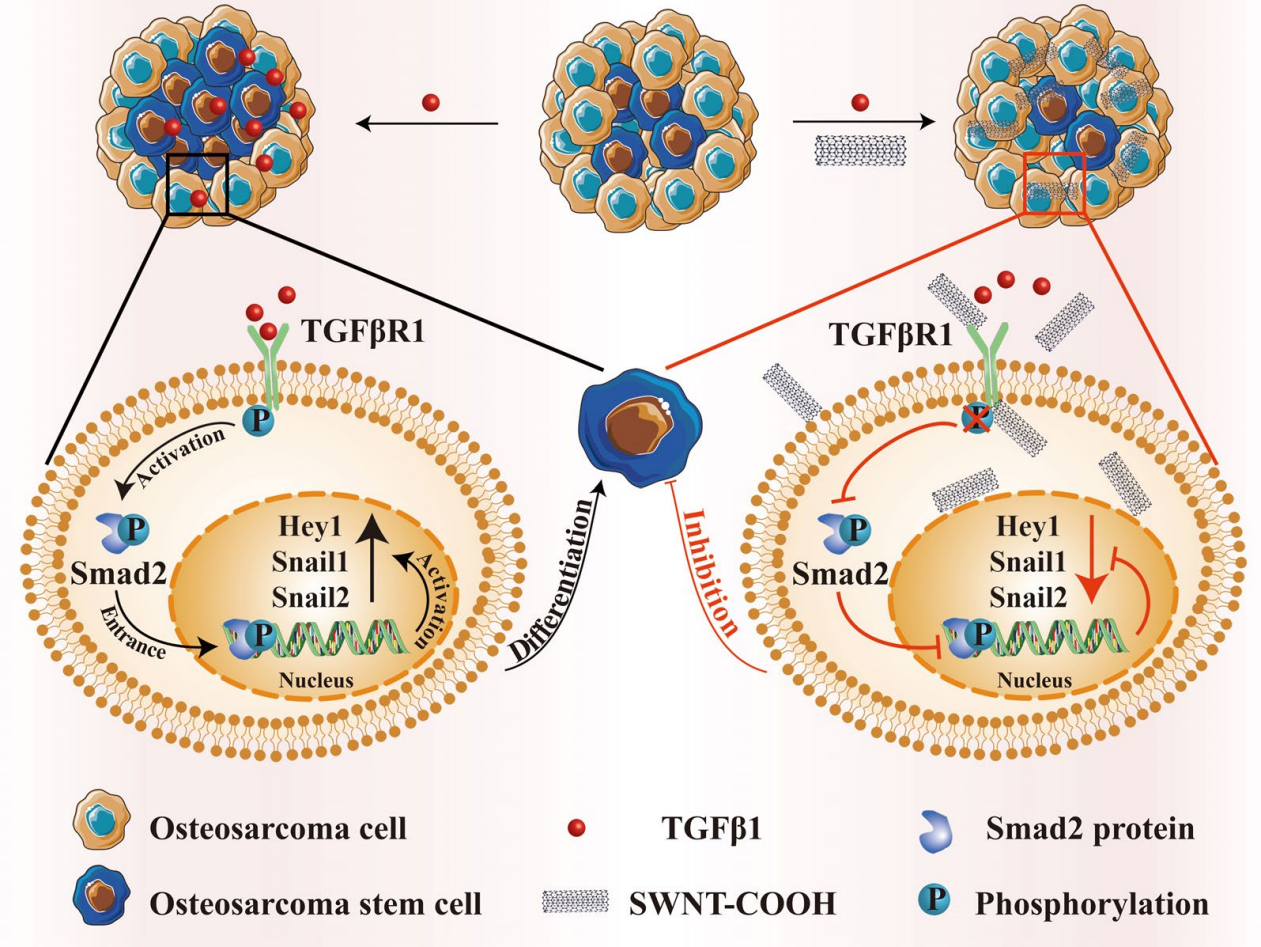
Schematic illustration of the working mechanism and therapeutic effects of SWNT-COOH on OSCs targeting

Osteosarcoma stem cells (OSCs) are a kind of CSCs that are very closely related to the metastasis and recurrence of osteosarcoma [121]. Transforming growth factor β type I (TGFβ1) is an important cytokine that contributes to the progression and carcinogenesis of osteosarcoma. Zhang et al*.* revealed that TGFβ1 signaling acted a pivotal role in maintaining the OSCs population and the high level of TGFβ1 in osteosarcoma microenvironment induced the differentiation of osteosarcoma cells into OSCs. Therefore, inhibition of TGFβ1 signaling was a possible solution to alter OSCs state and reduce their population [122]. Interestingly, an investigation by Miao et al*.* found that both raw SWNTs and carboxyl-modified SWNTs could specifically inhibit TGFβ1-induced differentiation of osteosarcoma cells, prevent the alteration of OSC phenotypes and attenuate the viability of OSCs under osteosarcoma microenvironment-mimetic conditions [75]. In addition, carboxyl-SWNTs were found to specifically bind to TGFβ1-induced activation of TGFβ type I receptor (TGFβR1) and suppress its downstream signaling, which drove OSCs formation and maintenance. All these observations indicated the unexpected biological effect of CNTs themselves to modulate OSCs, offering rational references for the CSCs-targeted therapy through the further application of CNTs-mediated treatment (Fig. 7).

#### Tumor vasculature targeting

Tumor vasculature targeting is an important therapeutic strategy for cancer treatment because tumor blood vessels can secrete a high level of pro-angiogenic factors that cause the formation of abnormal vascular networks to give rise to poorly perfused tumors [123]. Besides, tumor cells are able to produce a large number of hydrogen ions, lactate and pyruvate through anaerobic glycolysis, thus creating an acidic microenvironment [124]. These abnormalities of tumor vasculature build an intricate TME featured by hypoxia, acidity, and high IFP, which facilitate tumor progression, immunosuppression, and drug resistance [125]. Therefore, anti-angiogenesis or tumor vasculature normalization are potential approaches to prevent their growth and metastasis, exhibiting great research and clinical prospects in cancer treatment.

Compared to other carbon nanomaterials, CNTs, especially MWNTs, possess a better anti-angiogenic properties and can be applied as excellent inhibitors of blood vessel growth for tumor angiogenesis targeting [126]. Recent studies have also reported many cases on the application of CNT-based drug carriers against angiogenesis. For instance, Das et al*.* designed an injectable nanocarrier that combined *f*-CNTs with pH- and thermo-responsive polymer after rational modification [76]. Then, curcumin and DOX were loaded in this nanocarrier to obtain a synergistic antitumor effect. This nanocarrier achieved multidrug delivery with more than 90% drug encapsulation efficiency and a sustained drug release over 30 h under the stimulation of TME. Owing to the optical property of CNTs, a mild PTT effect could be simultaneously realized. Moreover, an outstanding antiangiogenic potential of this drug-loaded nanocarrier was firstly demonstrated ex-ovo by Chorioallantoic membrane Assay. In vivo results obtained from hepatocellular carcinoma (HCC)-bearing mice evidenced that these CNTs-involved carriers displayed an obvious effect on the reduction of MMP-9 which is closely related to cancer progression, implicating the potential of this nano carrier in combinational cancer treatment for anti-angiogenesis. Cheng et al*.* conjugated SWNTs with three different agents including the anti-angiogenesis drug thalidomide, cRGD peptide, and a fluorescent marker rhodamine [127]. They proved that *f*-SWNTs could target angiogenic sites and effectively inhibit angiogenesis in transparent zebrafish embryos and mammalian cells. In addition to chemical drugs, radiotherapy substances can also be attached to SWNTs and produce anti-angiogenesis effects. Ruggiero et al*.* modified SWNTs with radiometal-ion chelates and the tumor neovascular-targeting antibody E4G10 [128]. Through the guide of E4G10 antibody towards tumor angiogenic vessels, the radionuclide cargoes were specifically delivered to the tumor vasculature, after which the vessel image of colon adenocarcinoma tumor model was acquired by performing PET radio-immuno-imaging. Their study provided an alternative approach for tumor vessel targeting by combining SWNTs-based platform with radiotherapy and imaging.

Similar as SWNTs, MWNTs can also be functionalized to load different therapeutic agents to realize multiple effects for tumor vasculature targeting. Integrin α_ν_β_3_ has been extensively investigated as a potential target for cancer treatment due to its close relationship with angiogenesis. RGD peptide were reported to specifically bind with integrin α_ν_β_3,_ and thereby could be employed as targeting ligand for α_ν_β_3_-anchored delivery [129]. In our previous work, a co-delivery system based on MWNTs with dual-targeting ability was designed to aim at angiogenesis for lung cancer treatment [77]. iRGD peptide and candesartan were linked to PEI-modified MWNTs, which was then assembled with plasmid angiotensin II type 2 receptor (pAT_2_) through electrostatic interaction to form the final nanocomposites. Owing to the intracellular penetration ability of MWNTs, candesartan and pAT_2_ were successfully delivered into cytoplasm to achieve synergistic anti-angiogenic effects in A549 lung cancer cell, which also exhibited a significant suppression of tumor growth in A549 xenograft nude mice.

#### CNTs for immunotherapy

As is described previously, TME is a very complicated environment composed of parenchymal cancerous cells and stromal non-cancerous components. Immune cells, the most abundant cell types in TME, act a significant part in TME-based targeted therapy [130]. These immune cells dynamically interact with other components in TME and endow TME with immunosuppressive properties. Thus, targeting specific immune cells or modulating the immunosuppressive environment of TME present great significance in cancer immunotherapy. Owing to the feasible modification possibility of CNTs, a number of immunomodulators can be either directly connected on the surface of CNTs through functionalization or encapsulated into their inner structure for efficient delivery. In addition, it was reported that the systematic immune activity could be elevated by *s.c.* injection of water-soluble MWNTs in HCC tumor-bearing mice, which subsequently activated the complement system, activated the phagocytosis of macrophages, and facilitated the production of inflammatory cytokines, indicating the promising application prospect of CNTs in cancer immunotherapy [131]. By using combination of light energy, CNTs can also be applied as useful tools in phototherapy to effectively kill cancer cells, the released tumor-associated antigens (TAAs) and damaged-associated molecular patterns (DAMPs) through the induced immunogenic cell death (ICD) in target tumors can stimulate systemic immune response for enhanced immunotherapy effect [132]. Therefore, CNTs-mediated DDS displays a great research potential for precise immunotherapy.

Recently, Luo et al*.* designed a nanosystem composed of MWNTs-loaded ginsenoside Rg3 (Rg3-CNT) for triple-negative breast cancer (TNBC) treatment, which firstly demonstrated the new function of Rg3-CNT in elevating the therapeutic efficacy of Rg3 against TNBC through the inhibition of PD-1/PD-L1 axis, providing a potential strategy for TNBC treatment through immunotherapy [78]. In their study, Rg3-CNT was found to reduce PD-L1 expression and PD-L1 upregulation induced by IFN-γ in TNBC cells, meanwhile, PD-1 expression, PD-1/PD-L1 signaling were also suppressed, which remarkably inhibited the TNBC cell growth in vivo. Luo’s investigation offered new insights in revealing the correlation between Rg3-CNT and PD-1/PD-L1 signaling, indicating that the application of Rg3-CNT might be an alternative approach for TNBC immunotherapy. Another update study by Jin et al*.* showed that intratumoral delivery of immunoadjuvant CpG through CNTs could enhance the uptake of CpG in colon cancer cells, which remarkably inhibited primary tumor growth and liver metastasis [133]. In vitro results obtained from HCT116 human colon cancer cells demonstrated that CNTs-CpG complex could result in significant inhibitory effects on cancer cell growth, invasion and migration, implying the promising application potential of this nanocomplex for colon cancer immunotherapy. Similarly, Wang et al*.* used MWNTs to load CpG and DOX to achieve combinational PTT and chemo-immunotherapy for melanoma treatment [79]. Intratumoral injection of MWNTs-DOX and MWNTs-CpG followed by NIR irradiation contributed to a remarkable inhibition of tumor growth in vivo with an enhanced population of CD4^+^ and CD8^+^ T cells. In addition, this photo/chemo/immunotherapy synergistic strategy induced the shifting of TAM from M2 to M1, while reducing the amount of Tregs in TME, contributing to an elevated antitumor effect for melanoma treatment. Besides, various *f*-CNTs were reported to deliver vaccines like ovalbumin (OVA) or siRNA to protect them from degradation for enhanced immunotherapy effects, which shows great research interest and clinical value to treat many cancers [134–136].

#### Tumor microenvironment-responsive drug release

The conventional DDS based on EPR effect and receptor-mediated endocytosis often faces many limitations due to the biological complexity of TME where tumor cells live [137]. However, the hypoxic and acid features of TME with high levels of GSH and H_2_O_2_ enable the development of multistage TME-responsive drug release strategy. Triggered by various stimuli factors within TME, the carried drugs can be sequently released in a controlled or sustained profile to realize multiple therapeutic effects and deep tumor penetration. In addition, the reaction with these sensitive factors can alleviate tumor hypoxia and acidity, which creates a suitable environment for better therapeutic outcome. Therefore, the ongoing section highlights the recent examples about the smart drug delivery systems based on CNTs for TME-responsive drug release.

Yang et al*.* designed a kind of oxidized MWNTs with a large inner diameter to encapsulate anticancer drug cisplatin in their inner structure and to load DOX on the external surface [138]. PEG and folic acid were also employed to block the release of cisplatin from the inner of MWNTs. Consequently, the carried cargos showed a pH-sensitive release profile under pH 6.5, which mimicked the weak acidic TME, indicating the TME-responsive potential of this MWNTs-based nanosystem. Moreover, the antitumor efficacy tested on MCF-7 breast cancer cells showed that a more pronounced cytotoxicity was observed in the pH 6.5 condition than that in pH 7.4, confirming the best tumor killing effect of this nanosystem in acidic TME. Wang et al*.* constructed MWNTs-based nanosystem for MRI-guided and TME-responsive phototherapy [139]. A uniform MnO_2_ layer and photosensitizer Ce6 were modified on MWNTs for enhanced phototherapy. MnO_2_ could decompose H_2_O_2_ in the TME to produce ^1^O_2_ for promoting the photothermal effect generated by MWNTs, relieving tumor hypoxia, and facilitating Ce6-mediated PDT. In addition, MnO_2_ was able to rapidly deplete GSH in the acidic TME through redox reaction to reduce Mn^4+^ into Mn^2+^ ions, which augmented ROS-mediated cell death via chemodynamic therapy and accelerated Ce6 release, meanwhile, acting as a contrast agent for T1-MRI. Altogether, this multifunctional MWNTs-based platform offered a promising strategy to achieve synergistic cancer theranostics due to its TME-sensitive properties. Another case by Qin et al*.* showed that CNTs-based nanoplatform could also be encapsulated into a type of thermo/pH sensitive nanogel to achieve NIR-triggered, TME-responsive drug release [140]. The loaded DOX in this nanosystem showed a faster release rate at a higher temperature of 40 °C than 25 °C, meanwhile, more DOX released from this nanosystem at pH 5.0 than pH 7.4, which indicated that the combinational effects of photothermal effect mediated by CNTs upon NIR irradiation and acidic environment of TME were able to facilitate the release of DOX under dual stimuli.

## Carbon nanotubes in cancer diagnosis

**Fig. 8:**
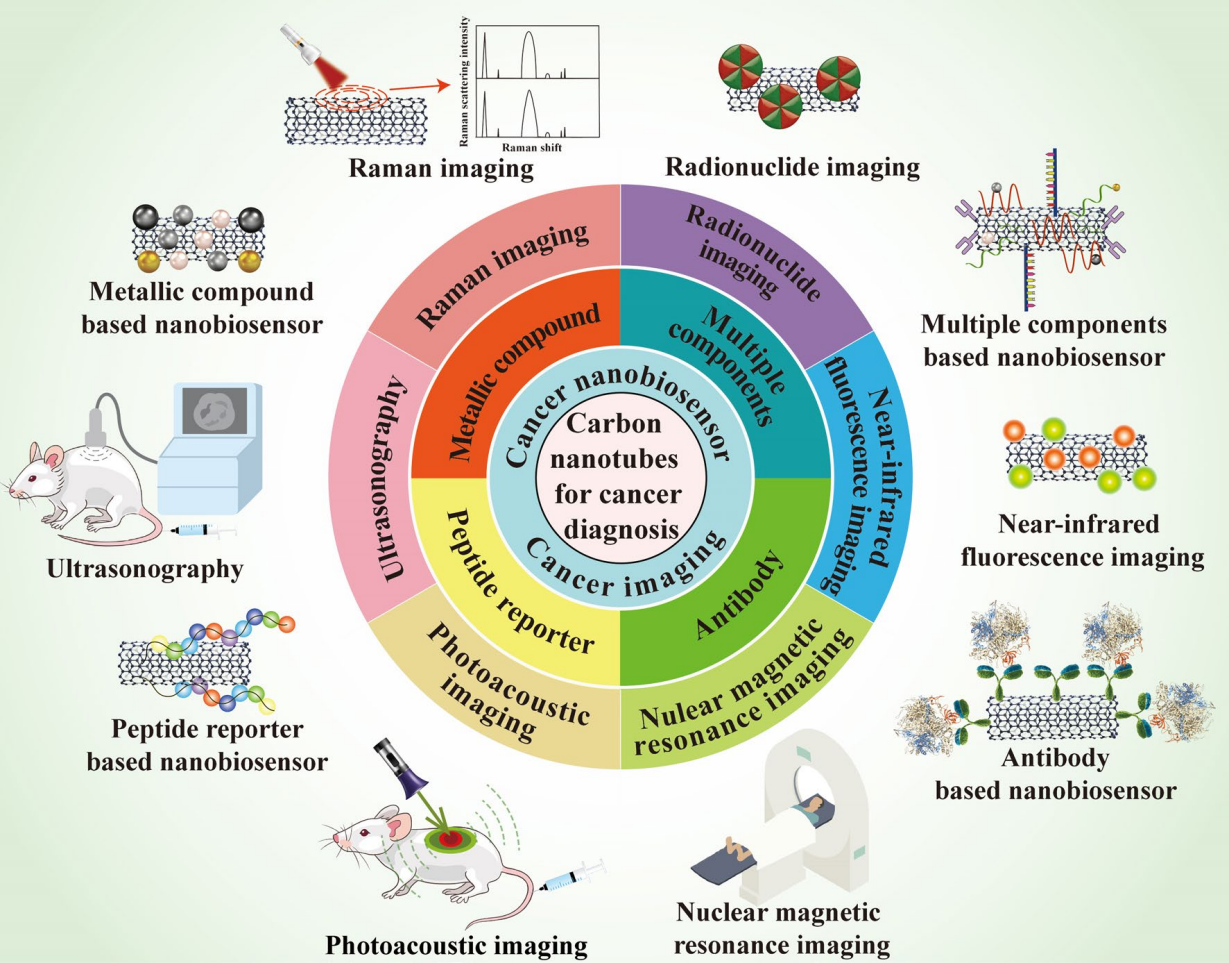
An overview of the contributions of CNTs to cancer diagnosis

Cancer diagnosis is a process of examining and detecting the etiologies and the related symptoms of different types of cancer through modern technologies. As a kind of inorganic nanomaterials with many unique properties, CNTs have arisen much attention in the field of cancer diagnosis. Therefore, in this section, the application of CNTs in various cancer imaging including Raman imaging, nuclear magnetic resonance imaging, ultrasonography, photoacoustic imaging, radionuclide imaging, near-infrared fluorescence imaging, as well as their contributions to cancer nanobiosensors will be discussed in detail (Fig. 8). Table 3 is a summary of all these CNT-based applications in cancer diagnosis.

**Table 3:** Summary of the application of CNTs in cancer diagnosis

Type of CNTs	Diagnostic methods	Effect	Refs.
SWNTs	Raman Imaging	Strong spots of Raman signals derived from the o-SWNTs-PEG in colon-26 cells can be obtained 5 days after administration	[141]
SWNTs	Raman Imaging	Possess a strong SERS effect suitable for labeling and fast Raman spectroscopic imaging of biological samples	[142]
SWNTs	MRI	Exhibit excellent MRI function for tumor diagnosis	[143]
MWNTs	MRI	Magnify the longitudinal proton relaxation process, possess T1 enhanced MRI effect	[144]
MWNTs	Ultrasonography	Show high contrast in ultrasonic imaging	[145]
MWNTs	Ultrasonography	Exhibit the ultrasound signal with strong, long-lived and high-quality properties after sonication treatment	[146]
SWNTs	PA Imaging	Shows obvious and stable PA signals	[147]
MWNTs	PA Imaging	Precisely target to the tumor site in vivo, strong PA imaging effect can be obtained	[148]
MWNTs	Radionuclide Imaging	Enable easy and direct SPECT/CT imaging	[149]
SWNTs	Radionuclide Imaging	Achieve efficient tumor tissue accumulation for the subsequent radionuclide imaging	[150]
SWNTs	NIRF imaging	Produces outstanding signal-to-noise performance and exhibits the high specificity towards the in situ ovarian tumor and the implanted tumor nodules	[151]
SWNTs	NIRF imaging	Obtain high-resolution intravital tumor vessel images through the thick skin in live mice	[152]
MWNTs	Nanobiosensor	Obtain high biosensing ability, and provide a wide linear range for detecting miR-21 with a very low detection limit in the early diagnosis of pancreatic cancer	[153]
SWNTs	Nanobiosensor	Exhibit broad detection range, low detection limit, and high specificity to only OPN during prostate cancer detection	[154]
MWNTs	Nanobiosensor	Achieve the simple detection of CDK1, and report the enzymatic activity of CDK1 for cancer diagnosis	[155]
MWNTs	Nanobiosensor	Possess high selectivity and sensitivity to HPV-18 for the early, rapid, easy, and accurate diagnosis of cervical cancer	[156]

### CNTs in cancer imaging

CNTs are multifunctional bio-probes with numerous unique properties, such as strong absorbance in NIR, good resonance Raman scattering, and high modifiability. Thus, CNTs can be regarded as a kind of suitable material for cancer imaging. Recently, the application of CNTs in cancer imaging has gained increasing attention in detecting various cancer types, which shows a promising research and clinical prospective in cancer diagnosis.

#### Raman imaging

Raman scattering is the transfer of emission wavelength of photons with light excitation. Due to the sharp electronic density of states at the van Hove singularities, CNTs exhibit numerous strong Raman peaks, including radial breathing model (RBM) and tangent G-module (TGM), which can be detected by a Raman microscope [157]. Sekiyama et al. constructed epoxide-type oxygen-doped SWNTs with the modification of PEG (o-SWNTs-PEG) as a recently developed over-thousand-nanometer (OTN)-NIR fluorescent probe for the investigation of the time-dependent change in OTN-NIR fluorescence images of colon-26 cancer cells [141]. Raman microscopy was adopted to study the o-SWNTs-PEG’s distribution in colon-26 cells. The experiment result showed that the Raman signals derived from this constructed nanostructure in colon-26 cells were enhanced evidently on the 5th day in contrast to the past, indicating the suitability of applying o-SWNTs-PEG in Raman imaging. Wrapping noble metal around CNTs’ surface is a common way for the enhancement of their Raman signals [158]. For this purpose, Wang et al*.* modified PEGylated CNTs with gold or silver to achieve the surface enhanced Raman scattering (SERS) effect of nanocomposites. After NIR irradiation, noble metal modified CNTs consumed the least time to acquire the Raman images compared with single CNTs [142].

#### Nuclear magnetic resonance imaging

MRI belongs to a non-invasive imaging technology without ionizing radiation damage to human body. It can obtain the original 3D cross-section images and shows great advantages in medical imaging [159]. Because CNTs can be applied as *T*_*2*_ spin dephasing contrast agents, they are common contrast agents that can be utilized to enhance nuclear magnetic resonance (MR) in clinic, and the MR images were generally obtained through an MRI scanner [160]. Yan et al*.* non-covalently combined NGR (asparagine-glycine-arginine) peptide with DOX and a type of MR contrast agent Gd-DTPA on CNTs. This nanosystem possessed both excellent antitumor efficacy and MRI function, exhibiting outstanding synergistic effect on tumor diagnosis and treatment [143]. Another research regarding the application of MWNTs in MRI was done by Zhang and teammates [144]. They synthesized a type of functionalized MWNTs (FA-GdN@CQDs-MWNTs/DOX) with the modification of gadolinium NPs (GdN), magnetofluorescent carbon quantum dots (CQDs), and folic acid (FA). These aforementioned modifications ensured outstanding stability and water stability of MWNTs, which are both essential for MRI. In vitro targeting MRI experiment result revealed that FA-GdN@CQDs-MWNTs magnified the longitudinal proton relaxation process, indicating the feasibility of applying this nano paramagnetic material to be a T1 contrast agent. Moreover, according to the in vivo experiment, the MR signal at tumor site was positively enhanced after *i.v.* administration, showing the tumor specificity for T1 enhanced MRI of this NPs.

#### Ultrasonography

Ultrasonography is a fundamental and popular diagnostic imaging technique due to the low detection cost and intrinsic safety [161]. Compared with other nano-based inorganic substances, CNTs are the most suitable nanomaterials for ultrasonic imaging due to the high signal produced by them during ultrasonography, and the signal could be detected by a contrast-enhanced ultrasound imager. Besides, the small size of CNTs enables them to cross the endothelial barriers, and subsequently to become a targetable material to reach tumor regions. Saghatchi et al. designed a kind of functionalized MWNTs that was modified with both magnetic Fe_3_O_4_ and gold NPs, obtaining *mf*-MWNT/AuNPs for cancer imaging and therapy [145]. According to the ultrasonic imaging effects, these NPs showed high contrast under all of the employed concentrations, exhibiting the possibility to become a type of contrast agent in ultrasonography. Moreover, Delogu et al. also carried out a study on MWNTs applied in ultrasonography [146]. In their research, MWNTs were firstly modified with azomethine ylides to improve their biocompatibility. According to various experiment results, these prepared functionalized MWNTs (ox-MWNT-NH_3_^+^) showed the ultrasound signal with strong, long-lived and high-quality properties after sonication treatment.

#### Photoacoustic imaging

PA imaging is a booming imaging technique which provides in vivo pathology with new characterization methods [162]. With the irradiation by pulsed laser, the absorbed luminous energy can be converted into ultrasonic through the thermal expansion of tissues and organs, and can subsequently be detected by sensors [163]. In contrast to other tumor diagnosis methods, the application of PA technology can improve the tissue penetration and the spatial resolution effectively. Because CNTs possess strong NIR absorption and deep tissue penetration, it is regarded as an ideal contrast agent for PA, and the PA signal could be recorded through a photoacoustic microscopy [164]. For example, Avti et al. directly detected, localized and measured the content of CNTs in different tissue samples through PA technology in cell imaging, and the signals of these samples were obvious and stable because of the powerful NIR absorbance of CNTs [147]. Besides, Wang and his teammates reported a kind of RGD-modified silica-coated, Au nanorods- functionalized MWNTs with active targeting ability for the in vivo PA imaging of gastric cancer [148]. They originally combined silica-coated Au nanorods (sGNRs) onto MWNTs, then attached RGD peptides to the sGNR/MWNTs nanostructure. After *i.v.* administration of this obtained RGD-conjugated sGNR/MWNTs probe, the treated mice were all diagnosed by an optoacoustic imaging system, which evidenced that this designed probe could precisely target to the tumor site in vivo to obtain strong photoacoustic imaging effect.

#### Radionuclide imaging

Radionuclide imaging is another type of imaging technology that is related to radioactive isotopes. Radiation source can form in vivo after the injection of radioactive chemicals such as ^111^In, ^131^I, ^64^Cu, and ^86^Y into the body and absorb subsequently human tissues and organs [165, 166]. The γ-ray released by in vivo radioactive isotopes during their decay process can be detected by nuclear detection devices in vitro, and the distribution density of the radioactive isotopes in vivo can be simultaneously obtained. In contrast to other imaging technologies, radionuclide imaging possesses both deep tissue penetration with negligible limitation and high sensitivity [167]. With the aim to investigate the organ biodistribution of various *f*-MWNTs in vivo, Wang et al. radio-labelled MWNTs with ^111^In to enable the in vivo single photon emission computed tomography/computed tomography (SPECT/CT) imaging [149]. With this radionuclide modification, detection of the *f*-MWNTs in vivo through whole body SPECT/CT imaging could be achieved easily and directly. ^131^I is another important radio isotope that can be applied in radionuclide imaging. Zhao et al. firstly modified SWNTs with polydopamine (PDA) and PEG, forming a nanostructure named SWNTs@PDA-PEG [150]. The functionalization of PDA shell enabled ^131^I to be labelled onto SWNTs@PDA-PEG for the subsequent radionuclide imaging. After administration, outstanding tumor tissue distribution of ^131^I-labelled SWNTs@PDA-PEG was observed in vivo through a gamma counter.

#### Near-infrared fluorescence imaging

Near-infrared fluorescence (NIRF) imaging is an exciting and fast progressing imaging technique, which can be applied in deep tissues and organs for cancer diagnosis. NIR biological window ranges from 780 to 1700 nm, and CNTs possess obvious optical absorption and intrinsic fluorescence in this range. Therefore, CNTs is a type of promising material which can be applied in NIRF imaging during cancer diagnosis, and the NIRF images are commonly acquired through an in-house in vivo imager [168, 169]. In contrast to MWNTs, SWNTs are more suitable for NIR imaging due to the higher absorbing properties with stronger optical absorption, better E11 optical transitions, and less photobleaching [170]. Ghosh et al. designed an M13-stabilized SWNTs probe which could precisely target to secreted protein, acidic and rich in cysteines (SPARC)-expressing tumor nodules in vivo [151]. Second-window NIR light (NIR-II) was applied in this research as the fluorescence source to circumvent optical scattering and obtain deeper tissue penetration effect during NIRF imaging. According to the diagnostic result, this NIR2-emitting SWNTs probes produced outstanding signal-to-noise performance and exhibited the high specificity towards the in situ ovarian tumor and the implanted tumor nodules existed on the surfaces of other peritoneal organs. Another research of adopting SWNTs to NIRF imaging was done by Welsher et al. [152]. In this research, SWNTs were firstly combined with phospholipid-PEG (PL-PEG) for the formation of biocompatible nanosystem with negligible destruction of the integrity of WNTs’ sidewall, which effectively enhanced the stability of SWNTs as well. With the application of an InGaAs camera, intravital tumor vessel images with high-resolution were obtained through the thick skin of the live mice made by the aforementioned functionalized SWNTs.

### CNTs in nanobiosensors

Biosensors were studied in 1962 by Clark and his teammates for the first time to qualify the glucose concentration combining electrochemical oxygen and glucose oxidase enzyme in aqueous media [171]. Recently, nanobiosensors have become more and more attentive mainly due to their easy application, fast response, and low cost [172]. Nanobiosensors are an important type of biosensors that usually consist of both biological recognition elements and nanomaterials with the dimensions range from 1 to 100 nm [173, 174]. Therefore, the events at nano-scaled level, such as the interaction between small molecules and specific receptors, can be monitored and detected by nanobiosensors sensitively. Due to the outstanding electrical conductivity, excellent electrocatalytic activity, high stability with slow oxidation kinetics and good modifiability, CNTs are a class of suitable inorganic material for the application as nanobiosensors in cancer diagnosis [175].

#### CNTs in combination with metallic compound

Some types of metallic NPs, like AuNPs and AgNPs, can be applied to modify CNTs for the construction of nanobiosensors. Rawashdeh et al. reported that AuNPs modified MWNTs were electrochemical nanobiosensors to detect a kind of micro ribonucleic acid named miR-21 [153]. The miR-21 derived from body fluid exhibits an important role in the early diagnosis of pancreatic cancer. However, the miR-21 existing in bloodstreams is generally at very low level during the early stage of pancreatic cancer, which brings great difficulty to cancer diagnosis [176]. With the application of AuNPs decorated MWNTs, the detection of miR-21 was greatly improved. According to the experiment result, this nanobiosensor was able to provide a broad linear range for detecting miR-21 with a detection limit as low as 3.68 femtomolar (fM) using the source measure unit (SMU). Thus, this nanobiosensor constructed by MWNTs possessed excellent application prospect in the early diagnosis of pancreatic cancer.

#### CNTs in combination with antibody

Nanoimmunosensors based on antibody are another kind of nanobiosensors for cancer diagnosis. Osteopontin (OPN) is a biomarker which can be applied to detect prostate cancer [177]. And the OPN level is important for predicting the survival time of prostate patient [178]. Traditionally, the measurement of OPN is usually achieved by ELISA assay, however, lack of sensitivity, high cost, and complex operation greatly hinder this detection method. Sharma et al. covalently combined OPN monoclonal antibodies onto the surface of carboxylated SWNTs, forming a nanoimmunosensor to determine OPN based on electrical detection for prostate cancer diagnosis [154]. This nanoimmunosensor exhibited high specificity to only OPN, which showed the outstanding selectivity during detection. Moreover, broad detection range and low detection limit can both be achieved with this nanoimmunosensor during prostate cancer detection.

#### CNTs in combination with peptide reporter

Peptide reporter modified nanobiosensors enable implementation to quantify peptide activity sensitively and dose-dependently in complex biological environments. CDK1 is responsible for the progression through mitosis and the coordination of G2/M transition, however, diagnostic methods for the detection and measurement of relative activities of CDK1 possess great difficulties due to the lack of sensitivity and specificity [179]. Tilmaciu et al. designed functionalized MWNTs that were modified with a kind of fluorescent peptide reporter which was specific to CDK1 for the sensitive and fluorescence-based quantification of CDK1’s activity through fluorescence imaging [155]. This nanobiosensor could not only achieve the simple detection of CDK1, but report the enzymatic activity of CDK1 as well. Therefore, the information on kinase function in the biological environment could be subsequently provided. And this nanobiosensor exhibited a promising future for the application in cancer diagnosis because CDK/cyclin activity could be detected in numerous cancer types and was associated with poor prognosis.

#### CNTs in combination with multiple modifications

In contrast to single modification, multiple modifications to CNTs can further improve the diagnostic effects obtained from the constructed nanobiosensors. Cervical cancer severely threatens women’s health and its incidence keeps growing at a very fast rate. Human papillomaviruses (HPVs), especially HPV type 18 (HPV-18), are important factors leading to cervical cancer [180]. Mahmoodi et al. designed a rGO-MWNTs/_L_-Cys-AuNPs nanocomposite through the modification of reduced graphene oxide (rGO), AuNPs, _L_-Cysteine (_L_-Cys), and single strand DNA (ssDNA) probe to MWNTs as an electrochemical DNA-biosensor to diagnose HPV-18 [156]. rGO improved the electrical conduction of this nanobiosensor, AuNPs not only increased the electrical conductivity, but immobilized DNA on the electrode surface as well. In addition, _L_-Cys also play an important role in immobilizing the ssDNA probes on the surface of this nanobiosensor [181]. According to their research, this nanobiosensor possessed high selectivity and sensitivity to HPV-18. Both the extracted DNA from HPV-18 patients and the synthetic DNA could be detected by this nanobiosensor in very small quantities with large detection range. Altogether, this constructed nanobiosensor could be regarded as an essential tool for the early, easy, rapid, and accurate diagnosis of HPV-18 compared with other classical detection methods.

## Conclusion and future prospect

This review gives a summary of the update advances of CNTs in cancer theranostics. Recent decades have witnessed the booming progress of nanotechnology in biomedical field. CNTs, one of the most widely investigated carbon-related nanomaterials, have gained considerable attention since their discovery. Because of their distinguish physiochemical properties and unique architecture like extensive surface areas, rich surface modification possibilities, high drug loading capacity and great optical features, CNTs can not only be adopted as fantastic transporters in drug delivery, but also occupy an important position in phototherapy-based cancer treatment. Through optimal functionalization, the inherent limitations of CNTs like unsatisfactory solubility, agglomeration and toxicity towards cells can be overcome, which improves their therapeutic performance in cancer treatment due to the enhanced biocompatibility and biodegradability, promoted tumor penetration ability and acquired active targeting capability. Moreover, many researches have demonstrated the multifunctional applications of CNTs in anticancer targeted drug delivery, which ranges from intracellular targeting spots to TME components, from single therapeutic modality to combinational treatment strategies, all indicating the promising future of CNTs in cancer treatment. In addition to their therapeutic applications, CNTs are also important participants in cancer diagnosis because of their peculiar characteristics. CNTs can be utilized in different cancer imaging like PA imaging, MRI, Raman imaging, radionuclide imaging and NIR fluorescence imaging. Besides, when combining with other diagnostic agents, CNTs are able to be employed in nanobiosensors for the early detection of various cancer types like pancreatic cancer, prostate cancer and cervical cancer with a high specificity. Therefore, CNTs are one of the versatile nanomaterials with broad biomedical application potential in both cancer treatment and diagnosis.

Despite the merits mentioned above, there are still many obstacles that impedes the clinical application of CNT-based therapies. For instance, the safety issue of CNTs in human body have not been fully studied. Although in vitro experiments carried out in many cell lines have demonstrated the safety of CNTs, in vivo tests based on animal models are often studied in a comparatively short period. Therefore, the long-term safety of CNT-based nanomedicine in human body still requires more investigations to confirm their application possibility in clinic. Besides, several functionalization methods of CNTs are relatively complicated in terms of large-scale production, thus, controllable industrial production with good reproducibility of functionalized CNTs is another problem that hinders their clinical translation. In contrast, another kind of carbon nanomaterials carbon quantum dots (CQDs) seem to perform better in terms of large-scale synthesis because of their relatively simply synthesis method, low cost and environmental-friendly property [182–184]. Unfortunately, CQDs also have concentration-dependent toxicity, which limits their application [185, 186]. Therefore,, in order to reduce the administration dosage to reduce toxicity, the accuracy of cancer cell targeting or TME components targeting through these carbon-based therapy should be evaluated and improved. From the aspect of diagnosis, graphene and its derivatives possess extraordinary electronic properties, which appears to be more promising in cancer biosensing [187–189]. However, although there are some difficulties that need to be solved, we are confident that CNTs are very promising nanotools with great research and clinical potential in cancer treatment and diagnosis. With the rapid progress in science and technology, more mature preclinical studies will be designed for CNT-based nanocarriers to provide us with more relevant date to explore their clinical possibilities.

## Data Availability

Not applicable.

## Abbreviations

5-FU: Fluorouracil
AgNPs: Sliver nanoparticles
Ago2: Argonaute-2
Anti-CD133: CD133 monoclonal antibody
Anti-HER2: HER2 monoclonal antibody
Anti-IGF1R: IGF1R monoclonal antibody
ATP: Adenosine triphosphate
AuNPs: Gold nanoparticles
Bax: Bcl-2-associated X
Bcl-2: B-cell lymphoma-2
Bcl-xL: B-cell lymphoma-extra large
CAFs: Cancer-associated fibroblasts
CAHA: Cholic acid-derivatized HA
CD133: Cluster of differentiation-133
CDK1: Cyclin-dependent kinases-1
Ce6: Chlorin e6
CNTs: Carbon nanotubes
CPT: Camptothecin
CQDs: Carbon quantum dots
cRGD: Cyclic RGD
CSCs: Cancer stem cells
DAMPs: Damaged-associated molecular patterns
DDS: Drug delivery system
DOX: Doxorubicin
DTPA: Diethylenetriaminepentaacetic acid
DWNTs: Double wall carbon nanotubes
ECM: Extracellular matrix
EGF: Epidermal growth factor
EGFR: Epidermal growth factor receptor
EPR: Enhanced permeability and retention
FA: Folic acid
fM: Femtomolar
GBM: Glioblastoma
Gd: Gadolinium
GO: Graphene oxide
GSH: Glutathione
HA: Hyaluronic acid
HCC: Hepatocellular carcinoma
hCE: Carboxylesterase enzyme
HepG2: Human hepatoma cells
HER2: Human endothelial receptor 2
HIF-1α: Hypoxia-inducible factor 1 alpha
HPVs: Human papillomaviruses
HRP: Horseradish peroxidase
ICD: Immunogenic cell death
IFN-γ: Interferon-γ
IFP: Interstitial fluid pressure
IGF1R: Insulin-like growth factor 1 receptor
IR: Infrared
LC: Liver cancer
mAbs: Monoclonal antibodies
MAPK: Mitogen-activated protein kinase
miR-21: MicroRNA-21
miRNA: Micro RNA
MMP-9: Matrix metalloprotein-9
MOMP: Mitochondrial outer membrane permeabilization
MRI: Magnetic resonance imaging
mtDNA: Mitochondrial DNA
MWNTs: Multi-wall carbon nanotubes
Near infrared: NIR
NGR: Asparagine-glycine-arginine peptide
NIR-II: Second-window near-infrared light
NIRF: Near-infrared fluorescence
OPN: Osteopontin
OSCs: Osteosarcoma stem cells
OTN-NIR: Over-thousand-nanometer near-infrared
OVA: Ovalbumin
p53: Oncogene suppressor protein 53
PAI: Photoacoustic imaging
pAT_2_: Plasmid angiotensin II type 2 receptor
PD-1: Programmed cell death protein 1
PDA: Polydopamine
PD-L1: Programmed cell death ligand 1
pDNA: Plasmid DNA
PEG: Polyethylene glycol
PEI: Polyethylenimine
PET: Positron emission tomography
Phe: Phenylalanine
PL: Phospholipid
PLGA: Polylactic-co-glycolic acid
Pt: Platinum
PtBz: Platinum (IV) prodrug
PTT: Photothermal therapy
RBM: Radial breathing model
rGO: Reduced graphene oxide
rHDL: Reconstituted high-density lipoprotein
Rho: Rhodamine-110
RISC: RNA induced silencing complex
RNAi: RNA interference
ROS: Reactive oxygen species
SERS: Surface enhanced Raman scattering
sGNRs: Silica-coated gold nanorods
SHG: Second-harmonic generation
shRNA: Short hairpin RNA
siRNA: Small interfering RNA
SMU: Source measure unit
Sor: Sorafenib
SPARC: Secreted protein, acidic and rich in cysteines
SPECT/CT: Single photon emission computed tomography/computed tomography
ssDNA: Single strand DNA
sSWNTs: Semiconducting single-walled carbon nanotube
SWNTs: Single-wall carbon nanotubes
T1-MRI: T1-magnetic resonance imaging
TA: Thermoacoustic shockwave
TAAs: Tumor-associated antigens
TAC: Tricarboxylic acid cycle
TAM: Tumor-associated macrophage
TAT: Transactivator of transcription
TGFβ1: Transforming growth factor β type I
TGFβR1: TGFβ type I receptor
TGM: Tangent G-module
TEM: Transmission electron microscope
TME: Tumor microenvironment
TNBC: Triple-negative breast cancer
TPEF: Two-photon excitation fluorescence
